# Reversing the great degradation of nature by reducing factors related to cropland expansion

**DOI:** 10.1073/pnas.2506601123

**Published:** 2026-05-18

**Authors:** Stephen Polasky, Erik Nelson, David Tilman, James Gerber, Justin Andrew Johnson, Erwin Corong, Forest Isbell, Jason Hill, Craig Packer

**Affiliations:** ^a^ Dept. Applied Economics, University of Minnesota, St. Paul, MN 55108; ^b^ Dept. Ecology, Evolution and Behavior, University of Minnesota, St. Paul, MN 55108; ^c^ Dept. Economics, Bowdoin College, Brunswick, ME 04011-8497; ^d^Project Drawdown, St. Paul, MN 55101; ^e^ Center for Global Trade Analysis, Department of Agricultural Economics, Purdue University, West Lafayette, IN 47907; ^f^ Dept. Bioproducts and Biosystems Engineering, University of Minnesota, St. Paul, MN 55108

**Keywords:** agricultural land requirements, crop yields, per capita food consumption, biodiversity, climate change

## Abstract

As one of the main causes of habitat loss, agricultural cropland expansion is a major threat to biodiversity. We analyze past and anticipated future trends in population, per capita crop demand, and crop yield to estimate agricultural cropland requirements globally by 2050 and 2100, assuming moderate levels of climate change. According to a model of “business as usual,” higher-income countries would be expected to show little or no net growth in cropland by the end of the century whereas cropland area could nearly double in lower-income countries. We consider two strategies to reduce global cropland expansion: decreasing per capita crop demand in higher-income countries by eating healthier diets, reducing food waste, and reducing biofuel production, and accelerating economic development in lower-income countries. Economic development in lower-income countries could reduce future cropland requirements via slower population growth, improved crop yield, and higher volumes of global crop trade, which could more than offset rising per capita crop demand. These impacts would far exceed reductions in cropland requirements from decreased crop demand in higher-income countries. Further, by combining accelerated economic development in lower-income countries with reduced crop demand in higher-income countries in tandem with reduced trade friction, global cropland requirements could shrink dramatically by the year 2100. Although economic growth is often considered to work in opposition to environmental conservation, accelerating economic development in lower-income countries would not only help alleviate poverty but could also confer benefits for biodiversity and global climate change.

## Significance

Rapid growth in global economic activity over the past century has reduced poverty and increased standards of living but has come at great environmental cost. However, our results suggest that accelerating economic development in lower-income countries could reduce future cropland expansion, benefiting biodiversity and climate mitigation through lower population growth, increased crop yields, and higher volumes of global crop trade, more than offsetting anticipated growth in per capita crop demand. The impact would be even greater if targeted development was coupled with reductions in crop demand in higher-income countries and expanded agricultural trade. Our findings provide a path to promoting multiple economic development and environmental sustainability outcomes.

The great acceleration in global economic activity since 1950 (1) has raised living standards and reduced poverty worldwide (2) but has simultaneously produced large declines in biodiversity (3) and rapid climate change (4). Agriculture is the dominant land use globally, with crops and pastures respectively covering ~12% and ~25% of the ice-free global land surface (5). Habitat loss from cropland expansion poses the greatest extinction threat to terrestrial species (6, 7), while being responsible for >10% of all anthropogenic GHG emissions (8, 9). Minimizing future agricultural land expansion would thus confer large biodiversity and climate-mitigation benefits (9–12).

Here we focus on crop production and demand factors that have led to past cropland expansion or contraction, and use these trends to illustrate potential trajectories of future cropland change to 2050 and 2100. We emphasize the contrasting trajectories between lower-income and higher-income countries, and their contribution to net cropland expansion.

Prior global-level analyses have suggested that closing yield gaps, shifting diets, and reducing food waste could minimize net cropland expansion (13–19). However, reducing food waste or shifting diets are extremely difficult to implement and require changes that much of the world appears unwilling to accept (20–22). In addition, our results suggest that reduced crop demand in higher-income countries has a much smaller effect on future cropland than economic development in lower-income countries. Other global-level analyses that more closely parallel this paper used general equilibrium and integrated assessment models to project future cropland area (23–28). These models incorporate a wide range of drivers of cropland area, including economic growth, yield change, population dynamics, climate change, and shifting demand patterns. However, their complexity makes it difficult to isolate and communicate the relative importance of various factors in driving cropland expansion and contraction. By contrast, our approach highlights in a clear manner the central role of economic development in lower-income countries in shaping future cropland needs and, ultimately, in securing a world that is less poor and more biodiverse.

We utilize a country-by-country crop production and demand accounting framework (29) measured in kilocalories (*kcal*) per year to estimate country-level cropland area requirements through 2100:

[1] Production=Yield×Cropland Area,

[2] $$\begin{array}{c} Consumption=Per\;capita\;consumption\times Population\times Ratio\\ \;of\;net\;exports, \end{array}$$

where the *Ratio of net exports* = $1+\frac{Exports-Imports}{per\;capita\;consumption\times Population}$. Equating crop production and demand and solving for cropland area generates the following expression:

[3] $$\begin{array}{c} Cropland\;Area\\ =\frac{Per\;capita\;consumption\times Population\times Ratio\;of\;net\;exports}{\mathrm{Yield}}. \end{array}$$

Eq. (**3**) shows that a country’s total cropland area increases with higher per capita crop demand, population size, and net exports, and decreases with higher yields. Per capita crop demand includes all crop uses (direct human food consumption plus crop waste, crops grown for livestock, aquaculture, biofuels, and other plant-based products). Expansion of cropland occurs when total crop demand (per capita crop demand × population × ratio of net exports) grows faster than yields. Increasing net exports leads to increases in cropland expansion in exporting countries but reductions in cropland expansion in importing countries.

The components of crop production and demand vary with a country’s economic development status, so we base our quantitative analyses on World Bank classifications of income groups (low, lower-middle, upper-middle, high) as of 2018 (30) (*SI Appendix*, *Supplementary Materials and Methods* and Table S18). We treat China and India separately because of their size and unique socioeconomic trajectories.

## Results

### Historical Drivers of Cropland Expansion.

**Fig. 1.**
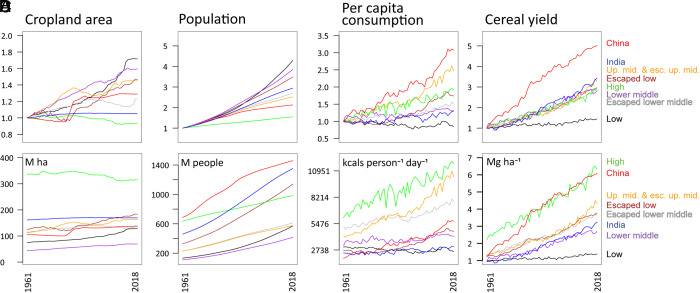
Changes in: (*A*) Cropland area, (*B*) Population, (*C*) Per capita crop demand (kcal per person per day), and (*D*) Cereal yield, 1961–2018, by income group plus China and India. *Top*: changes relative to 1961 (1961 = 1). *Bottom*: group totals/averages. Figures only include information for countries with complete data from 1961–2018 and exclude former Soviet Union countries and other countries with significant border changes between 1961 and 2018 (see *SI Appendix*, Text 5 and Table 18 for the list of countries). We use cereal yields as more complete data are available, and cereal yields have been shown to be a good proxy for overall yields (33). Data from FAO (5). See *SI Appendix*, Texts 4 and 5 and Table 18 for more details.

In low-income countries, cropland area has almost doubled since 1961 (Fig. 1*A*) as the population has increased ~300% (Fig. 1*B*), while per capita crop demand declined slightly (Fig. 1*C*), a worrisome trend given widespread malnutrition in many poor countries (31), and cereal yields remained virtually unchanged (Fig. 1*D*). We present yields for cereals instead of all crops because the data for cereals are more complete going back to 1961, and available evidence shows high correlation between the trends in cereal yield and all-crop yields (32, 33). In contrast, total cropland area has declined in high-income countries since 1961 (Fig. 1*A*), coincident with relatively small increases in population and large increases in cereal yields (Fig. 1 *B* and *D*). The correlation between yield and economic development has been long recognized in the agricultural development literature, though whether higher yields lead to higher incomes, or higher incomes lead to higher yield, or both, is less clear (34–38). The decline in cropland area in high-income countries likely would have been even greater except for large increases in per capita crop demand (Fig. 1*C*) owing to a shift toward grain-fed meat and crop-based biofuels.

These drivers of cropland change are also seen in the historical records in individual countries. For example, as the United States underwent rapid economic development from 1866 to 1920, harvested cropland expanded with its growing population, but cropland area stabilized over the following century despite continued growth in population as rapid yield increases exceeded growing demand (*SI Appendix*, Text 6 and Fig. S9). Similarly, China experienced rapid population growth and cropland expansion from 1961 to the 1990s while classified as a low-income country. As China rose to upper-middle income status after the 1990s, cropland expansion ceased (39) due to slowing population growth (Fig. 1*B*), rapidly increasing yields (Fig. 1*D*), and increasing imports that offset rising per capita crop demand (Fig. 1*C*).

While the aggregated data in Fig. 1 illustrates striking patterns across income groups, countries vary considerably within each income group (*SI Appendix*, Fig. S11). We quantified the relative contribution of population, per capita crop demand, yield, exports, and imports to each country’s area of cropland with country-level data for the years 1961 to 2016 and present the results by income group in Table 1 (40). Population growth is widely considered to be a major factor driving expansion of cropland in developing countries (41), and we find that population has the largest relative impact of any variable on cropland expansion across all income groups. Per capita crop demand also exhibits a positive and statistically significant relationship with cropland area across all income groups. Although higher crop demand in the low-income countries ameliorates malnutrition of millions of people (42, 43), per capita crop demand in high-income countries has increased to levels that far exceed caloric needs.

**Table 1.** Estimated relationships between cropland area and population, per capita demand, cereal yield, and net exports 1961 to 2016

Independent variables	All countries	Low and escaped low-income	Lower-middle and escaped lower-middle income	Upper-middle, escaped upper-middle, and high-income
log population (billions; β_1_)	0.763*** (0.085)	0.464** (0.190)	0.651*** (0.201)	0.882*** (0.155)
log per capita daily kcal demand (β_2_)	0.351*** (0.059)	0.419*** (0.070)	0.299*** (0.099)	0.318*** (0.073)
log cereal yield (Mg ha^−1^; β_3_)	−0.298*** (0.044)	−0.246*** (0.044)	−0.243*** (0.073)	–0.369*** (0.071)
log export value (2010 USD; β_4_)	0.065*** (0.017)	0.026 (0.016)	0.081*** (0.020)	0.094*** (0.026)
log import value (2010 USD; β_5_)	−0.022 (0.02)	–0.029 (0.022)	−0.103*** (0.038)	−0.002 (0.049)
Observations (N x T)	6,258	2,120	2,047	1,979
Within R^2^	0.455	0.355	0.377	0.523

*Notes*: For all model estimates, the dependent variable is the natural log of cropland area. All model estimates include country and year-fixed effects. When estimating these relationships, each country is weighted by the amount of land rated as highly or moderately suitable for rainfed production of cereals with low level of inputs as of 2005 (40). “***” indicates an estimated coefficient with a *P*-value <0.01, “**” 0.01 < *P* < 0.05, and “*” 0.05 < *P* < 0.10. Estimated coefficient SE (in parentheses) are clustered at the country-level. “N” = number of countries and “T” = number of time periods. The regression includes only countries with complete data over the entire time frame 1961–2016 and excludes countries with population less than one million (see *SI Appendix*, Text 5 and Table 18 for the list of countries). “Within R^2^” indicates the degree of within-country variation in cropland explained by the model. Data on cropland area, population, demand, cereal yields, and exports/imports come from FAO (5). See *SI Appendix*, Text 4 for more details.

**Fig. 2.**
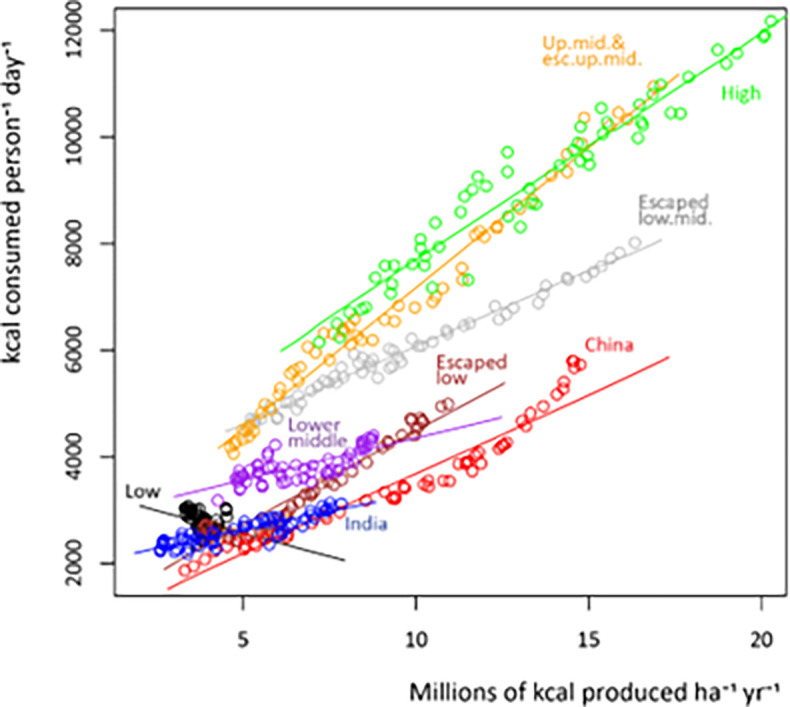
Relationship between crop yields and per capita crop demand by income group 1961–2018. The relationships show a nearly linear positive correlation between the trend in crop production and demand for all income groups except the low-income countries. Figures only include information for countries with complete data from 1961–2018 and exclude former Soviet Union countries and other countries with significant border changes between 1961 and 2018 (see *SI Appendix*, Text 5 and Table 18 for the list of countries). Data from FAO (5). See *SI Appendix*, Texts 4 and 5 for more details.

Higher yields have been proposed as a solution for preventing land clearance (44–46) and indeed higher yields are associated with lower cropland area (Table 1). However, the estimated coefficient on yield is smaller in absolute magnitude than the estimated coefficients on population or per capita crop demand for all income groups (except for per capita crop demand in high-income countries). Increased yields tend to lower prices and hence lead to greater crop demand rather than being used in full to reduce cropland area [consistent with literature on rebound effects (47, 48)]. Our data suggest that between 60 to 80% of increased yield goes to meet increased demand while only 20 to 40% is available for decreasing cropland area. Per capita crop demand has grown in every income group that has shown substantial increases in crop yields (Fig. 2), as governments have generally supported agricultural interests by encouraging demand through alternative uses for crops, such as livestock feed and biofuels, rather than cropland retirement (20, 49).

The relationship between cropland area and exports is positive for all income groups and statistically significant for all income groups except the low and escaped low-income category (50) (Table 1). The relationship between cropland area and imports is negative for all income groups but is only statistically significant for the lower-middle and escaped lower-middle-income category.

### Future Land Requirements.

Future cropland requirements in each country will be affected by future population, per capita crop demand, trade flows, yields, climate change, and other factors. We consider four scenarios that illustrate the key drivers of future cropland requirements and show a wide range of potential outcomes: i) Business-as-usual (BAU), ii) Reduced per capita crop demand in higher-income countries, iii) Accelerated economic development in lower-income countries, and iv) Equitable development that combines ii) and iii). To highlight the potential impacts of trade, we also consider *Frictionless trade* scenario in which crops are grown in the most efficient locations and shipped to where they are needed to meet demand. We use IPCCs Representative Concentration Pathway (RCP) 4.5 to incorporate future climate change, as changing climate is expected to have impacts on yields (see *SI Appendix*, Text 3.3 on yields). Details on population, per capita crop demand, yields, trade, and climate change are provided in the *Materials and Methods* and *SI Appendix*, Texts 2 and 3 and Tables S1–S17 and Figs. S1–S8.

**Fig. 3.**
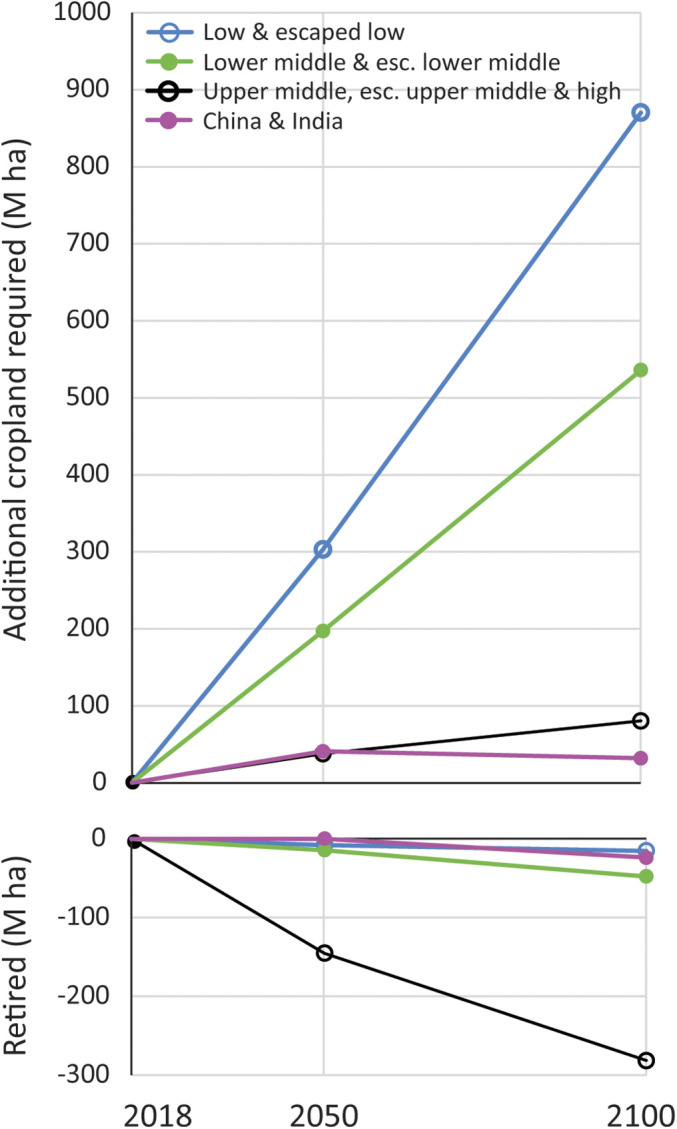
Additions/reductions in land for agricultural crop production required by 2050 and 2100 for each World Bank economic classification according to the BAU scenario. We aggregate all country-level cropland additions for each economic classification (*Top*) separately from all reductions for that same classification (*Bottom*).

**Fig. 4.**
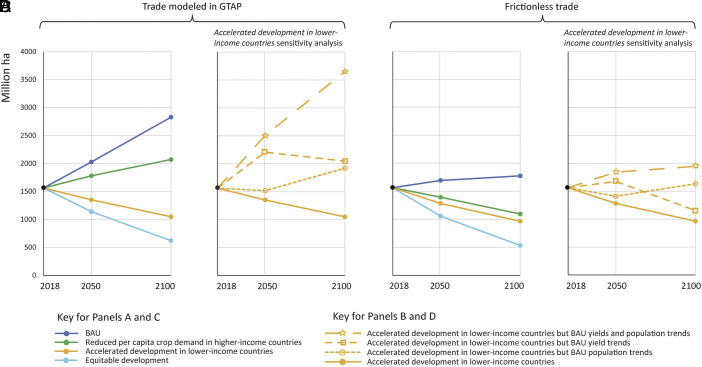
Total hectares of cropland required by 2050 and 2100. Data are calculated on a country-by-country basis and then aggregated to a global level for four scenarios: *BAU*, *Reduced per capita crop demand in the higher-income countries*, *Accelerated development in the lower-income countries*, and *Equitable development* that combines reduced crop demand in higher-income countries with accelerated development in lower-income countries. (*A*) Cropland requirements under each scenario with projected patterns of global trade according to GTAP. (*B*) Cropland requirements under *Accelerated development* assuming various combinations of *BAU* yields and population growth. (*C*) Cropland requirements under each scenario in the case of *Frictionless trade*. (*D*) Cropland requirements under *Accelerated development* with *Frictionless trade* with the same combinations of *BAU* as in *B*.

*Business-as-usual (BAU):* Considerable cropland expansion is projected to occur in low-income, escaped low-income, lower-middle-income, and escaped lower-middle-income countries (hereafter “lower-income countries”) because growth in crop demand is projected to outpace growth in yields (Fig. 3). In stark contrast, substantial areas of existing croplands in the upper-middle-income, escaped upper-middle-income, and high-income countries (hereafter “higher-income countries”) could be retired by 2050 and 2100 (Fig. 3). Though land retirements in higher-income countries could provide global environmental benefits, these benefits would be dwarfed by the far greater losses of biodiversity and carbon emissions from expansion of agricultural land in lower-income countries. Globally, the ~1,560 million ha of cropland currently in production worldwide are predicted under BAU to expand by an additional ~1270 million ha by 2100 (Fig. 4*A*).

*Reduced per capita crop demand in higher-income countries:* In 2018, daily per crop capita crop demand in higher-income countries averaged over 11,500 kcal, four times the minimum required for a healthy diet. This high crop demand has been largely driven by grain-fed meat/dairy products and biofuels (51). Shifting consumer preferences toward plant-rich diets in wealthier parts of the world would reduce land pressures (43, 52) and confer environmental and health benefits (44, 53–56). Replacing biofuels with renewable energy (wind, solar) used in electric vehicles would also reduce the demand for cropland. For example, in the United States over a third of maize is converted to ethanol (57). Limiting daily per capita crop demand in higher-income countries as of 2100 to the observed 2018 global average (5,000 kcal) would allow the retirement of considerable cropland in higher-income countries. However, cropland requirements in lower-income countries would continue to expand, leading to global land requirements expanding by ~507 million ha by 2100 (Fig. 4*A*). The impacts of reduced crop demand in the higher-income countries could potentially be more meaningful if it was coupled with a coordinated international policy of “land-sparing” agricultural trade (see *Frictionless trade* below).

*Accelerated economic development in lower-income countries:* Faster economic development in lower-income countries would raise per capita income, which is correlated with lower population growth consistent with the demographic transition (Fig. 1*B*), higher per capita crop demand (Fig. 1*C*), and higher yields (Fig. 1*D*). Rising standards of living increase demand for educated employees, educational opportunities for females, women’s empowerment, availability of family planning, urbanization, and declining reliance on labor-intensive agriculture, all of which can contribute to declining birth rates and lower population growth (58, 59). Rising standards of living can also increase yields through access to new technology, improvements in manufactured and human capital that increase productivity (34–38). Besides helping hundreds of millions of people out of poverty and improving living conditions, the overall impact of *Accelerated economic development in the lower-income countries* would result in a sizable net reduction in global cropland requirements by 2050 and a reduction of ~513 million ha by 2100 (Fig. 4*A*). These results occur because of reduced population growth and increased yields associated with rising incomes, which could outpace increases in per capita crop demand in the lower-income countries.

The *accelerated economic development in lower-income countries* scenario involves three changes relative to *BAU*: greater per capita consumption, lower population growth, and higher yield, in lower-income countries. To assess the relative importance of their potential impacts on global cropland area, we ran additional analyses with various permutations of these three factors (Fig. 4*B*). First, global cropland requirements would be increased well beyond BAU if *accelerated economic development* were only to increase consumption in lower-income countries. Second, if *accelerated development* in lower-income countries were to increase consumption and ameliorate population growth, crop area would rise in 2050 then fall slightly by 2100. Third, if *accelerated economic development* only affected consumption and yields, crop area would remain relatively constant until 2050 then rise to a similar level in 2100 as seen in the previous permutation. Slower population growth and increased yields would make similar contributions to reducing crop area by 2100. Thus, even if our future projections are unrealistic, improvement in either population growth or crop yields could still produce substantial reductions in future cropland requirements.

*Equitable development:* A more comprehensive approach to limiting cropland expansion would be two-pronged: reducing per capita crop demand in the higher-income countries while simultaneously accelerating economic development in the lower-income countries. In this scenario, global agricultural cropland could be substantially reduced (Fig. 4*A*). Although it may be impossible to fully achieve such a rapid reduction in crop demand in higher-income countries or acceleration in development in lower-income countries, our analysis suggests that even partial success in one or both dimensions could achieve substantial benefits for climate, biodiversity, and global food security.

*Frictionless trade:* Another strategy for reducing future global cropland requirements would be through expanded food exports from high-yield higher-income countries (60). *Frictionless trade* is defined here as having crop production occur in locations where yields are highest with trade meeting the demand in each country. *Frictionless trade* would magnify the impacts of reduced crop demand in higher-income countries as their production would then be available to meet the increased crop demand of lower-income countries, and, since yields in higher-income countries tend to be larger than in lower-income countries, such trade allows meeting crop demand with less total cropland. With *Frictionless trade*, global croplands might only need to expand by ~211 million ha by 2100 under *BAU* and could decline if combined with *Reduced per capita crop demand in the higher-income countries* or with *Accelerated development in lower-income countries* (Fig. 4*C*). *Frictionless trade* also reduces the importance of yield growth in lower-income countries, shown by the small gap between *Accelerated development in lower-income countries* with and without accelerated yield growth, because exports from higher-income countries would be able to meet demand in lower-income countries (Fig. 4*D*). However, increased reliance on agricultural trade raises obvious risks to food security and employment. In times of shortage, countries will first feed their own populations, leaving importing countries to scramble for alternatives (61). Greater agricultural trade could also disrupt economic activity in importing countries. In 2018, 59.6% of the workforce in lower-income countries was employed in agriculture vs. 3.1% in higher-income countries (62). Note that *Frictionless trade* would have much less impact in combination with *Accelerated development in lower-income countries* or *Equitable development* because lower-income countries would produce far more of their own needs in these two scenarios.

## Discussion

Accelerated economic development in lower-income countries could decrease or even prevent habitat loss from cropland expansion in some of the most biodiverse countries in the world. Although economic growth is often considered to work in opposition to conservation, higher incomes could help close yield gaps and lower population growth in the lower-income countries to an extent that would likely exceed the increased cropland requirements from rising per capita crop demand. A similar point has been made regarding global carbon emissions (63). Increasing per capita food crop demand is itself a desirable outcome in any country with extensive malnutrition. Increased yields have long been central concerns of national governments, farmers, and foreign assistance (36, 64), and targeted development projects can make substantial impacts. One large aid program increased yields by >50% over a 20-y period in target sub-Saharan African countries, whereas yields remained essentially unchanged without comparable investment (65). Research increases productivity in agriculture (66–68), and countries such as Brazil, China, and India have invested in research and development programs over the past few decades that greatly raised their agricultural productivity (69).

On its own, any reform that reduces current per capita demand in higher-income countries would have little effect on global biodiversity, unless it is linked with expanded food exports from higher-income countries with stable or declining populations to lower-income countries with still-expanding populations. However, increased reliance on international food trade runs obvious risks of disruption, whereas efforts to improve the self-reliance of the lower-income countries would reduce their dependence on food imports.

Although intensive agriculture can create its own environmental problems, modern methods of sustainable intensification can substantially reduce negative ecological impacts (70–72) and cause less harm to biodiversity compared to the conversion of previously uncultivated land (73–76). The ideal economic development policy should incorporate meaningful conservation measures that not only minimize environmental impacts from agricultural intensification (77) and expand investment in agricultural research and development but also address ancillary issues such as increased demand for bushmeat in wealthy urban centers (78).

Decelerating cropland expansion has even greater positive impacts on biodiversity when accounting for the movements of land into and out of crop production within each country, as demonstrated by satellite data available from 1992 (79). The environmental impacts of habitat destruction from cropland expansion far exceed the gains from retiring cropland in the short- to medium-term (80, 81). Agriculture within many countries has expanded into natural habitat while preexisting croplands have been abandoned, thus masking substantial habitat destruction from land turnover (*SI Appendix*, Text 7 and Fig. S10; also see *SI Appendix*, Fig. S9*C* for the United States). In addition, switching among crops, such as between perennial and annual crops, and the recent increase in tropical oil crops, also has major environmental impacts (82).

Achieving accelerating economic development, increasing agricultural research and development spending, reducing crop demand in higher-income countries, and reducing trade barriers all require overcoming difficult obstacles. For example, replacing biofuels with alternative energy sources would reduce crop demand in higher-income countries (83), but this would be likely to face political opposition. Agricultural policy is replete with entrenched subsidies that have detrimental environmental impacts (84, 85). The imposition of tariffs and recent cuts to international aid to the world’s poorest nations could potentially make even our BAU scenario unrealistically optimistic.

However, movements toward accelerated economic development and increasing yields in lower-income countries along with reduced crop demand in higher-income countries and expanded trade could be truly transformative in terms of biodiversity, climate change, human health, and well-being. Such a transformation could make major contributions to attaining the UN Sustainable Development Goals, the UN Convention on Biological Diversity’s targets of protecting and restoring 30% of the earth’s surface for nature by 2030 and the Paris climate accords by reducing agricultural-based greenhouse gas emissions.

## Materials and Methods

### Historical Drivers of Cropland Expansion.

We quantify the relative contribution of population, per capita crop demand, yield, exports, and imports to each country’s area of cropland using a logged version of the crop production-demand accounting framework shown in Eq. (**3**) with country-level data for the years 1961 to 2018:

[4] $$\begin{array}{c} logA_{\mathrm{it}}=\beta_{P}logP_{\mathrm{it}}+\beta_{C}logC_{\mathrm{it}}+\beta_{Y}logY_{\mathrm{it}}+\beta_{E}logE_{\mathrm{it}}+\beta_{I}logI_{\mathrm{it}}\\ +\alpha_{i}+\tau_{t}+\varepsilon_{\mathrm{it}}, \end{array}$$

where $A_{\mathrm{it}}$ is cropland area in country *i* in year *t*, $P_{\mathrm{it}}$ is population (billions), $C_{\mathrm{it}}$ is per capita annual crop demand (kcal capita^−1^), $Y_{\mathrm{it}}$ is cereal yield (Mg ha^−1^ on harvested cropland), $E_{\mathrm{it}}$ is the value of agricultural exports (2010 USD), $I_{\mathrm{it}}$ is the value of agricultural imports (2010 USD), $\alpha_{i}$ is a fixed effect for country *i*, $\tau_{t}$ is a fixed effect for year *t*, and $\varepsilon_{\mathrm{it}}$ is a normally distributed error term. We use country and year fixed effects to control the impact of a country’s time-invariant and time-specific factors. Export and import data are reported in monetary terms rather than in kcals, so we include separate terms for exports and imports rather than the net export ratio as shown in the crop production-demand accounting framework in Eq. (**3**).

### Future Land Requirements.

We calculated cropland area (Eq. **3**) for each country in the following four scenarios: i) *BAU*, ii) *Reduced per capita crop demand in higher-income countries*, iii) *Accelerated economic development in lower-income countries*, and iv) *Equitable development*, in the years 2050 and 2100. In each scenario we use IPCC’s Representative Concentration Pathway (RCP) 4.5 and the Global Trade Analysis Project computable general equilibrium model to estimate impacts of future conditions on crop yields and trade. We also analyze each scenario with an assumption of *Frictionless trade* based on balancing global crop production and demand (see below). We summarize the assumptions for each scenario below (and in *SI Appendix*, Table S1).

#### Population.

In the *BAU* and *Reduced per capita crop demand* scenarios (*SI Appendix*, Table S1) we used the United Nation’s (UN’s) 2050 and 2100 median population forecast for each country (*SI Appendix*, Text 3.1 and Table S2) (86). Assuming rapid attainment of the UN Sustainable Development Goals, Vollset et al. (2020) projects a population of 6.3 billion in 2100 versus the UN median projection of 10.7 billion (87). We used 2050 and 2100 country-level population estimates from Vollset et al. (2020) in the *Accelerated economic development* and *Equitable development* scenarios (*SI Appendix*, Text 3.1 and Table S2).

#### Per capita crop demand.

To determine country-level estimates for annual per capita crop demand in 2050 and 2100, we use data from 1988 to 2018 to estimate the growth in per capita GDP as a function of per capita GDP (*SI Appendix*, Text 3.2). In the *BAU* and *Reduced per capita crop demand* scenarios, we use this relationship to project per capita GDP for each country. In the *Accelerated economic development* and *Equitable development* scenarios, we used an increased growth equation for countries with GDP per capita less than $8,100 (*SI Appendix*, Text 3.2 and Table S3 and Figs. S1–S4). We then estimated the relationship between real per capita GDP (2010 USD) and per capita daily crop demand at the income group-level for 2014–2018 (*SI Appendix*, Text 3.2 and Fig. S5 and Table S4). We project per capita crop demand based on this relationship and projected per capita GDP in 2050 and 2100.

#### Yields.

We estimated country-level crop yield in kcals per hectare harvested for the years 2050 and 2100 for each of our four scenarios under RCP 4.5 (*SI Appendix*, Text 3.3). We used data from UN FAO (5), USDA NASS (88), and Brazil’s Instituto Brasileiro de Geografia e Estatística (89) and the methods and data of Ray et al. (90–92) to compile a 1980 to 2018 panel of administrative unit-level yields and harvested hectares for 23 focus crops, which supply the vast majority of produced kcals (*SI Appendix*, Tables S5-S7). This dataset is normalized so that it agrees with FAO data at the national level. We first estimated country-level crop yield in kcals per hectare harvested for the years 2050 and 2100 without considering the effects of climate change by assuming a linear extrapolation of observed crop yields subject to the constraint that extrapolated yields do not exceed a modeled yield ceiling, as described in the next paragraph. We then adjusted future yields assuming moderate climate change (RCP 4.5) (see *SI Appendix*, Text 3.3.4 for details on climate adjustments).

Following Gerber et al. (93), we used quantile regression techniques and the panel of administrative unit-level yields, temperature patterns, precipitation, and soil, topography, and irrigation conditions, to estimate a yield ceiling function for each administrative unit with each country, measured in tons per hectare, for each of the 23 focus crops (*SI Appendix*, Texts 3.3.1–3.3.3 and Tables S8–S10 and Fig. S6). We use linear extrapolation at each administrative unit to project yield ceilings into the future. To ensure that yield ceilings stay bounded, we hold yield ceilings constant past 2050. For the *BAU* and *Reduced per capita crop demand in higher-income countries*, we extrapolate past yield trends going forward until 2100 with an adjustment for climate change, or until the yield ceiling for that country is reached. We maintain these assumptions for wealthier countries (countries with GDP per capita above $8,100) in the *Accelerated economic development in lower-income countries* and *Equitable development* scenarios. For poorer countries (countries below $8,100 GDP per capita) we assume more rapid growth in yield in the *Accelerated economic development in lower-income countries* and *Equitable development* scenarios, where yield is set equal to the maximum of twice the 2018 yield (subject to not exceeding yield ceilings) or the average of projected yield and yield ceilings. We justify this more rapid growth for the 32-y period between 2018 and 2050 based on the observed more than doubling of cereal yields between 1986 and 2018 in five countries that successfully escaped low-income status (Bangladesh, Bhutan, Cambodia, Laos, and Vietnam) (5). For 2100, we assume that yield in lower-income countries will equal the average of 2050 yield and the yield ceilings. Details on yield data and modeling are described in *SI Appendix*, Text 3.3 and Tables S8–S13, and Fig. S6–S8. Note that our sensitivity analysis for accelerated economic development in Fig. 4 *B* and *D* includes a scenario with BAU yield increases, as the escaped low-income countries in south Asia may not reflect the potential performance of their counterparts in the rest of the world.

#### Trade.

We projected country-level net trade in agricultural commodities in 2050 and 2100, measured in USD, using the Global Trade Analysis Project (GTAP) model version 7.0 (94) and the GTAP Database version 11 (95) (*SI Appendix*, Text 3.4). We ran a disaggregated version of the GTAP model, including 33 sectors and 37 countries, with 2017 as the base year. To generate country-level net trade volumes for 2050 and 2100 we used our country-level projections for 2050 and 2100 population, real annual per capita GDP, per capita daily crop demand, and yield projections assuming RCP 4.5. Given these inputs, the GTAP model predicted country-level net exports (in billions of real 2017 USD) in 2050 and 2100 for each socioeconomic scenario (*SI Appendix*, Table S1). We converted country-level net exports in dollars to net exports in kcals using country-level relationships between net exports in dollars in 2017 and kcals in 2018 (*SI Appendix*, Text 3.4 and Tables S14−S17).

In the *Frictionless trade* scenario, we calculated how much cropland would be needed to match global crop production and demand without trade restrictions. For 2050 and 2100, we aggregated country level crop demand to generate global crop demand. We also aggregated country level production with yields in 2050 and 2100, assuming 2018 cropland area. We then expand or contract cropland area to match global crop production with demand assuming global average yield (*SI Appendix*, Text 3.4.1).

## Supplementary Material

Appendix 01 (PDF)

## Data Availability

Data used for the analysis in this paper are available at https://doi.org/10.5061/dryad.59zw3r2df (96). All study data are included in the article and/or *SI Appendix*.
