# Supplementary material for: Reversing the great degradation of nature by reducing factors related to cropland expansion
Source: Proc Natl Acad Sci U S A. 2026 May 18;123(21):e2506601123. doi: 10.1073/pnas.2506601123 (PMC13214005; doi:10.1073/pnas.2506601123)
Supplement: Supplementary file 1 — Appendix 01 (PDF) [file pnas.2506601123.sapp.pdf]

Supplementary Materials and Methods
SM Texts 1 to 8
SM Figures 1 to 11
SM Tables 1 to 18

Supplementary Materials and Methods References

Supplementary Materials and Methods

SM Text 1. Projecting country-level cropland area

In the main text, we developed a crop production-demand identity measured in kilocalories (kcals) per year:

$$\underbrace{Production}_{kcal} = \underbrace{Yield}_{kcal\ ha^{-1}} \times \underbrace{Cropland\ Area}_{ha} \quad \text{(SM1)}$$

$$\underbrace{Consumption}_{kcal} = \underbrace{Per\ capita\ crop\ demand}_{kcal\ person^{-1}} \times Population \times Ratio\ of\ new\ exports \quad \text{(SM2)}$$

where the $Ratio\ of\ new\ exports = 1 + \frac{(\overbrace{Exports}^{kcals} - \overbrace{Imports}^{kcals})}{Per\ capita\ crop\ demand \times Population}$.

Equating crop production and demand and solving for cropland area yields equation (3) shown in the main text:

$$Cropland\ area = \frac{Per\ capita\ crop\ demand\ x\ Population\ x\ Ratio\ of\ net\ exports}{Yield} \quad \text{(SM 3)}$$

The land area that balances crop production and demand in year t in a country is a function of four country-level inputs: 1) per capita crop demand in year t, 2) population in year t, 3) ratio of net exports in year t, and 4) yield in year t. The trajectory of these inputs across countries over time varies according to the economic scenarios described below. All data comes from FAO [main text ref. 5]. Production and demand data include crops grown for livestock and biofuels. Area used for pasture or hay production and kcals generated by pasture or hay are not accounted for in our model.

SM Text 2. Scenarios

We project country-level cropland area needed in 2050 and 2100 under four scenarios: *business-as-usual* (*BAU*), *Accelerated development in low-income countries*, *Reduced per capita crop demand in high-income countries*, and *Equitable development* that combines accelerated economic development in low-income countries with reduced per capita crop demand in high-income countries. Within each scenario we assume moderate climate change (Representative Concentration Pathway (RCP) 4.5). Climate directly affects crop yields and indirectly affects trade flows (described below). The assumptions for each scenario are summarized in SM Table 1.

SM Table 1. Summary of scenarios

		**Business-as-usual**	**Accelerated development in lower-income countries**	**Reduced per capita crop demand in higher-income countries**	**Equitable development**
Population	Country-level population projections source	UN median population forecast[66]	Sustainable development population forecast[42]	UN median population forecast[66]	Sustainable development population forecast[42]
	2050 Global total	9.6 billion	8.6 billion	9.6 billion	8.6 billion
	2100 Global total	10.7 billion	6.3 billion	10.7 billion	6.3 billion
Per Capita Crop Demand: Projections of kcal per person per day based on relationship with GDP per capita (equation 1 below)	2050	Per capita crop demand as a function of BAU GDP per capita in 2050 (equation 2 below)	Per capita crop demand as a function of accelerated GDP growth per capita in 2050 (equation 3 below), with the average of projected trend and 5,000 kcals per person per day for countries with per capita crop demand below 5,000 kcals per person per day.	Per capita crop demand as a function of BAU GDP per capita in 2050 (equation 2 below) with the average of projected trend and 5,000 kcals per person per day for countries with per capita crop demand above 5,000 kcals/ day.	Per capita crop demand as a function of accelerated GDP growth per capita in 2050 (equation 3 below), with the average of projected trend and 5,000 kcals per person per day for countries with per capita crop demand above 5000 kcals/day, and the average of projected trend and 5,000 kcals per person per day for countries with per capita crop production below 5,000 kcals/day.

		Business-as-usual	Accelerated development in lower-income countries	Reduced per capita crop demand in higher-income countries	Equitable development
	2100	Per capita crop demand as a function of BAU GDP per capita in 2100 (equation 2 below)	Per capita crop demand as a function of accelerated GDP growth per capita in 2100 (equation 3 below), with a minimum of 5,000 kcals per person per day.	Per capita crop demand as a function of BAU GDP per capita in 2100 (equation 2 below) unless the projected per capita trend exceeds 5,000 kcals/day. In these cases, per capita crop demand is set at 5,000 kcals/day..	5,000 kcals per capita per day for all countries
Yield	2050	Projection of 1988-2018 trends with climate change subject to not exceeding yield ceilings.	For lower-income countries with accelerated growth in GDP per capita (per equation 3 below), the maximum of twice the 2018 yield or the average of projected yield and the yield ceiling, subject to not exceeding the yield ceiling. For all other countries, BAU yield scenario.	Projection of 1988-2018 trends with climate change subject to not exceeding yield ceilings.	For lower-income countries with accelerated growth in GDP per capita (per equation 3 below), the maximum of twice the 2018 yield or the average of projected yield and the yield ceiling, subject to not exceeding the yield ceiling. For all other countries, BAU yield scenario.
	2100	Projection of 1988-2018 trends with climate change subject to not exceeding yield ceilings.	For lower-income countries with accelerated growth in GDP per capita (per equation 3 below), the average of 2050 yield and the yield ceiling. For all other countries, BAU yield scenario.	Projection of 1988-2018 trends with climate change subject to not exceeding yield ceilings.	For lower-income countries with accelerated growth in GDP per capita (per equation 3 below), the average of 2050 yield and the yield ceiling. For all other countries, BAU yield scenario.
Trade	GTAP modeled trade	Net exports of kcals from each country were created with GTAP using scenario-specific country-level yields, GDP per capita, and population, (methods described in SM Text 3.d.)			
	Frictionless trade	Crop production occurs in locations where yields are highest, with trade occurring to meet demand in each country			

	Business-as-usual	Accelerated development in lower-income countries	Reduced per capita crop demand in higher-income countries	Equitable development
(1) Per capita crop demand based on the relationship with GDP per capita	$ln\ daily\ kcal\ cons\ cap^{-1}\ (t) = 6.431988 + 0.240283 \times \ln\left(GDP\ cap^{-1}\ (t)\right)$			
(2) BAU growth of GDP per capita	$\frac{d\left(GDP\ cap^{-1}(t)\right)}{dt} = GDP\ cap^{-1}(t) \times$ $\left[-0.002589 + 0.00344 \times \ln\left(GDP\ cap^{-1}(t)\right) - 0.00321\left(\ln\left(GDP\ cap^{-1}(t)\right) - 7.991\right)^2\right]$			
(3) Accelerated growth of GDP per capita	All countries with per capita income above \$8,100 growth according to BAU GDP per capita growth as shown in (2). Country with GDP per capita below \$8,100 grow according to: $\frac{d\left(GDP\ cap^{-1}(t)\right)}{dt} = GDP\ cap^{-1}\ (t) \times [0.16 - 0.015 \times \ln\left(GDP\ cap^{-1}\ (t)\right)]$ Once per capita GDP reaches \$8,100, the GPD per capita growth trajectory is BAU.			

SM Text 3. Inputs: Population, Daily per capita kcal Crop Demand, Yield, and Trade
a. *Population*
In the *BAU* and *Reduced per capita crop demand in high-income countries* scenarios, we use the United Nation's (UN's) median population forecast for each country in 2050 and 2100 [main text ref. 86]. In the *Accelerated development in low-income countries* and *Equitable development* scenarios, we use country-level 2050 and 2100 population estimates from Vollset et al. (2020) [main text ref. 87]. For most countries, Vollset et al. (2020) project much lower populations in 2050 and 2100 than the UN median population estimate. Globally, Vollset et al. (2020) projects 8.6 billion people by 2050 versus the UN's prediction of 9.6 billion people. By 2100 the absolute difference in predictions is even greater, 6.3 billion versus 10.7 billion. Country-level population projections in Vollset et al. (2020) are noticeably lower than those of the UN in low-income countries, which significantly reduces pressure on croplands in low-income countries. Vollset et al. (2020)'s alternative results are explained by their expectations for 1) greater economic development around the world than assumed by the UN, which is consistent with our *Accelerated development in low-income countries* and *Equitable development* scenarios, and 2) deeper cuts in total fertility rates in response to economic growth than assumed by the UN. SM Table 2 contains country-level population for 2018, and country-level population projections for 2050 and 2100 under each scenario.

SM Table 2: Population by country

Country	***BAU* and *Reduced per capita crop demand in high-income countries* (UN median population projections)**			***Accelerated development in low-income countries* and *Equitable development* (Vollset et al. 2020)**		
	**2018**	**2050**	**2100**	**2018**	**2050**	**2100**
Afghanistan	33948445	64682976	74937961	34064581	56967905	42718040
Albania	2774543	2424052	1088338	2784894	2540406	1318433
Algeria	41259179	60923390	70704619	41341121	57435905	60072244
Angola	29086326	77420355	188283132	29175426	48164146	46709368
Argentina	44688411	54867248	56802493	44846086	51263958	39620879
Armenia	3031480	2816114	2038613	3044990	2669085	1334464
Australia	24256771	32814106	42876774	24336782	31398468	31057483
Austria	8819917	9130698	8676844	8858038	8624043	6006086
Azerbaijan	10322527	11064995	9192141	10358458	10462334	5750026
Bahrain	1502422	2316047	2251891	1504064	2119366	1543680
Bangladesh	158454579	192567779	151393018	158884971	165959038	74194976
Belarus	9473309	8634216	7429673	9531134	7880614	4912815
Belgium	11383128	12220769	12492831	11434228	12677486	11569480
Belize	402337	571048	620439	403245	550778	443294
Benin	11928882	24280484	47208998	11968587	19176949	17451128
Bhutan	970369	904614	686287	972384	1166957	715261
Bolivia	11770099	15839561	17390956	11801865	16614158	15821484
Bosnia and Herzegovina	3389813	2685037	1641054	3407537	2663124	1188909
Botswana	2319633	3509813	4165861	2326649	3182828	2780756
Brazil	213517322	228980400	180682762	214170215	229751667	151638736
Brunei Darussalam	438021	492391	389884	438936	498913	299850
Bulgaria	6998785	5385444	3588337	7049986	4943188	2278672
Burkina Faso	21771608	43432174	83194233	21863305	36079522	34250565
Burundi	11239480	25324770	50904072	11281494	19100818	14890560
Cambodia	16362178	21860917	21355011	16411883	19438501	13136914

Country	*BAU* and *Reduced per capita crop demand in high-income countries* (UN median population projections)			*Accelerated development in low-income countries* and *Equitable development* (Vollset et al. 2020)		
	**2018**	**2050**	**2100**	**2018**	**2050**	**2100**
Cameroon	28435163	50573036	90225178	28528301	41400836	32838670
Canada	36316131	45669453	56953224	36447572	42033190	37061622
Central African Republic	4660422	8400954	11631410	4691592	4381122	1172911
Chad	15804659	34031310	61849820	15878713	30375451	32906552
Chile	18068311	20319303	17332335	18121634	19597570	13431890
China	1417784383	1402405167	1064993457	1422831298	1266112553	699740670
Colombia	51188078	55957823	45221276	51300909	60005708	42357942
Congo Dem. Rep.	83218424	194488663	362031082	83534189	130481151	106119954
Congo Rep.	4999994	10701991	20961987	5018619	6104595	3917176
Costa Rica	4703223	5772686	4797969	4714454	5336565	3501199
Cote d'Ivoire	25650727	51263670	96632721	25741965	38642702	35064490
Croatia	4253456	3364799	2183314	4277739	3125571	1220890
Cuba	11364345	10162395	6671461	11412675	9273457	4065617
Cyprus	1271433	1354589	1308911	1275135	1331942	760382
Czech Republic	10598921	10545590	10274070	10651465	9436744	6038931
Denmark	5754638	6245374	6872907	5780133	5970084	4883320
Djibouti	1141342	1295368	1331656	1144458	1454452	914491
Dominican Republic	10568607	12795809	11012548	10600838	11210210	5522041
Ecuador	16908284	23316455	24482822	16949683	20450430	14544651
Egypt Arab Rep.	98126557	159956809	224735180	98368406	141478869	163923800
El Salvador	6114297	6936792	4766079	6134063	5386547	1062603
Equatorial Guinea	1390229	2820923	4510631	1393726	2254795	2085513
Eritrea	5978758	6005492	9061777	5999326	7855040	4904652
Estonia	1311767	1158412	837720	1319054	1120266	696786
Eswatini	1143549	1704428	2145198	1148554	1517848	1204191
Ethiopia	106061121	205410671	294392903	106325118	173762916	153929458
Finland	5533218	5486152	5254370	5559096	5542570	4588289
France	65966412	67586729	65497773	66235445	68954527	60131133
Gabon	1733145	3809049	5859225	1738567	2243907	1595836
Gambia The	2184855	4882099	8175553	2191504	3135420	2257889
Georgia	3681555	3516870	2514130	3705499	3091267	1845012
Germany	83446235	80103973	74740596	83891127	81572858	60061620
Ghana	30853965	52016123	79011409	30953163	43424373	36292295
Greece	10380948	9029254	6583364	10437481	8711708	4726209
Guatemala	17246609	26920714	31270073	17289737	22542908	17510096
Guinea	12136633	25971807	45256876	12189465	17804606	14111089
Guinea-Bissau	1906430	3557202	5706289	1914118	2792768	2232339
Guyana	747723	824958	530598	750411	731866	289942
Haiti	12019250	14877785	14760450	12062653	14181986	8193308
Honduras	9693482	13830802	14325309	9714781	13511357	11181959
Hungary	9697710	8470232	6856920	9755988	7974029	4530702
Iceland	339973	376683	353291	341004	385471	323129
India	1394649837	1639176036	1447025612	1399466407	1537938676	929867647

Country	*BAU* and *Reduced per capita crop demand in high-income countries* (UN median population projections)			*Accelerated development in low-income countries* and *Equitable development* (Vollset et al. 2020)		
	**2018**	**2050**	**2100**	**2018**	**2050**	**2100**
Indonesia	260417420	330904672	320782426	261215858	291719429	202691132
Iran Islamic Rep.	83020889	103098083	98587985	83203692	92844873	62227448
Iraq	44424512	70940131	107711273	44498641	69463666	73249453
Ireland	4899466	5677620	5684962	4914043	5581339	4815048
Israel	9092548	12720415	18127523	9113938	13265828	17651515
Italy	60516123	54381674	39992910	60805483	51359749	27791291
Jamaica	2779694	2960321	1793106	2789357	2383452	705989
Japan	127979216	105804023	74959378	128614122	100984888	52685740
Jordan	10866545	12932404	13644110	10879688	16054441	14131975
Kazakhstan	18122849	24024036	27917815	18188062	22488524	23243394
Kenya	49386922	91575092	125423855	49529471	72240852	59252122
Korea Dem. People's Rep.	25745823	26561614	22792833	25859897	22867900	11009591
Korea Rep.	52849188	46829924	29541953	52993096	49325031	24721258
Kuwait	4331917	5393471	6188983	4335694	5494755	4154525
Kyrgyz Republic	6466839	9126098	10984938	6483194	8082367	6033045
Lao PDR	7083823	9479753	8423978	7106140	8538337	5446805
Latvia	1926828	1479176	1114401	1940191	1217343	342417
Lebanon	8618081	6528162	5707209	8634033	10638102	6564965
Liberia	4841446	9339755	15525036	4856624	6902236	4999777
Libya	6995790	8525412	8011540	7012552	8491290	6991290
Lithuania	2831898	2121388	1524086	2851338	2183269	1236690
Madagascar	26913532	54048131	99957340	27004175	43869988	42216661
Malawi	17675129	38142971	66559386	17738503	28566095	25731961
Malaysia	31043095	40550371	40078167	31123363	40197523	35133136
Mali	20915719	43585533	80382697	21008801	35782463	34824142
Mauritania	4017824	9024891	17064607	4027467	6408943	6303886
Mexico	128320178	155150814	141509942	128659864	153763626	107617452
Moldova	3712822	3360176	2011568	3733073	2919970	1266388
Mongolia	3302870	4448931	5387208	3313283	4170165	4405658
Montenegro	626454	589336	453656	629559	553760	296749
Morocco	35829772	46165497	44707864	35936467	40875030	28435596
Mozambique	30764837	65312931	123646947	30890420	43481819	32079807
Myanmar	53258262	62253423	55298929	53457959	62055695	45815802
Namibia	2396228	3981125	5373589	2404573	3610780	3752584
Nepal	30255092	35323925	23708193	30343430	32773583	15553093
Netherlands	17068170	17165370	15759617	17139597	16298355	11150403
New Zealand	4490618	5607793	6008350	4505973	5282287	4966098
Nicaragua	6487426	8531045	8124405	6498495	7514354	3836615
Niger	22226381	65593043	164947337	22312896	45873233	57813679
Nigeria	212381781	401314997	732941596	213167895	375065096	408539598
Norway	5311600	6600338	7952927	5330830	6503172	6468506
Oman	4656896	6915451	7267893	4662393	7388354	6562913
Pakistan	218465211	338013194	403102827	219158562	277071740	179876941
Panama	3978768	5852939	6439999	3987670	5257227	4996203
Papua New Guinea	9453980	14204060	19782704	9496651	13367253	11369601
Paraguay	7028919	9101631	8733633	7044635	8887154	6961596

Country	*BAU* and *Reduced per capita crop demand in high-income countries* (UN median population projections)			*Accelerated development in low-income countries* and *Equitable development* (Vollset et al. 2020)		
	**2018**	**2050**	**2100**	**2018**	**2050**	**2100**
Peru	33814631	40373987	39158180	33881963	45640377	39341452
Philippines	105222500	144488171	146327441	105542910	135446031	107028821
Poland	38343548	33294573	23032569	38531576	31193799	13655426
Portugal	10647820	9084579	6984955	10701182	8617373	4157834
Qatar	2797052	3851121	4162408	2798902	3399297	1884146
Romania	19320618	16259784	11877961	19445035	14515460	6601505
Russian Federation	146144318	135824486	126142651	147026993	128745267	89370500
Rwanda	12895207	23048001	33412643	12929704	21029354	19573686
Saudi Arabia	34975153	44562484	42231012	35021014	42819789	27304292
Senegal	15082629	33186850	63515357	15125401	22804856	18789250
Serbia	8824555	7083830	4217425	8881866	6917234	3180010
Sierra Leone	8024625	12944875	16675197	8059463	11392952	8033582
Slovak Republic	5420217	4983647	3829211	5445389	4594285	2289604
Slovenia	2069380	1939598	1676264	2078830	1783237	991202
Somalia	17380980	34922449	75716429	17455997	25663629	6589623
South Africa	55649272	75517909	79190832	55882468	70036227	60360210
Spain	46419116	43637408	33209856	46612980	41275382	21541780
Sri Lanka	21676860	21813964	15275209	21737607	19814606	7307846
Sudan	41327689	81192821	142342356	41427936	59782641	51155668
Suriname	576764	680234	613122	578840	615571	389134
Sweden	10113605	11389198	13022532	10156203	11389921	10722551
Switzerland	8667701	9817716	11041105	8697762	9598700	7390159
Syrian Arab Republic	18118322	33129325	36103178	18173773	18684458	10147067
Tajikistan	9458797	16208246	25328026	9482454	12783904	8203774
Tanzania	55624021	129386845	285651846	55792661	93352458	88792083
Thailand	70806164	65940494	46015553	71033603	65643718	33204004
Togo	7686042	15415505	26949191	7710958	10642718	7895732
Tunisia	11544822	13796600	12972285	11576408	13044437	9930024
Turkey	81491070	97139565	86170462	81684824	103035787	86104696
Turkmenistan	5050735	7949276	8421349	5066828	6317156	5944170
Uganda	40392845	89446901	136784876	40507377	72310682	73348515
Ukraine	44401778	35219046	24413452	44730639	32269016	14741951
United Arab Emirates	9821717	10425284	12909869	9834809	8694541	3148682
United Kingdom	66994646	74081983	78053263	67279929	72601285	63743105
United States	326844607	379419097	433853891	328195093	349966209	285593626
Uruguay	3433055	3639106	3181955	3448905	3419742	2269554
Uzbekistan	32703931	42942489	42270842	32802157	40125492	34377216
Venezuela RB	31219896	37023121	34240724	31302524	35729338	24484095
Vietnam	97003379	109605010	97437286	97296105	102894466	62759041
Yemen Rep.	31297406	48080012	53171320	31378046	45002075	30699204
Zambia	17881703	39120906	81546198	17939627	29937547	30216815
Zimbabwe	15060463	23947921	30965421	15118394	24511258	27237303

b. Annual kilocalorie crop demand per capita

We use a three-step process to determine country-level estimates for annual kcal crop demand per capita in 2050 and 2100. First, we generate per capita GDP projections for each country in 2050 and 2100. Second, we estimate the historic relationship between per capita GDP and per capita daily kcal crop demand to generate a projection of per capita daily kcal crop demand in each country for 2050 and 2100. Third, we multiply projected daily kcal crop demand per capita levels by 365.25 to create projected per capita annual kcal crop demand levels.

To project a country's real annual per capita GDP in 2050 and 2100 (2010 USD) under the *BAU* and *Reduced per capita crop demand* scenarios we fit an equation that described the annual change in a country's real per capita GDP from 1988 to 2018 based on the current level of per capita GDP [main text ref. 2]:

$$\frac{d\left(GDP\ cap^{-1}(t)\right)}{dt} = GDP\ cap^{-1}(t) \times \left[-0.002589 + 0.00344 \times \ln\left(GDP\ cap^{-1}(t)\right) - 0.00321 \times \left(\ln\left(GDP\ cap^{-1}(t)\right) - 7.991\right)^2\right] \quad \text{(SM 4)}$$

We set annual change in that country's real GDP per capita to 0 in cases where the model predicts negative economic growth (a possibility when real annual GDP per capita is very low; see SM Fig. 1).

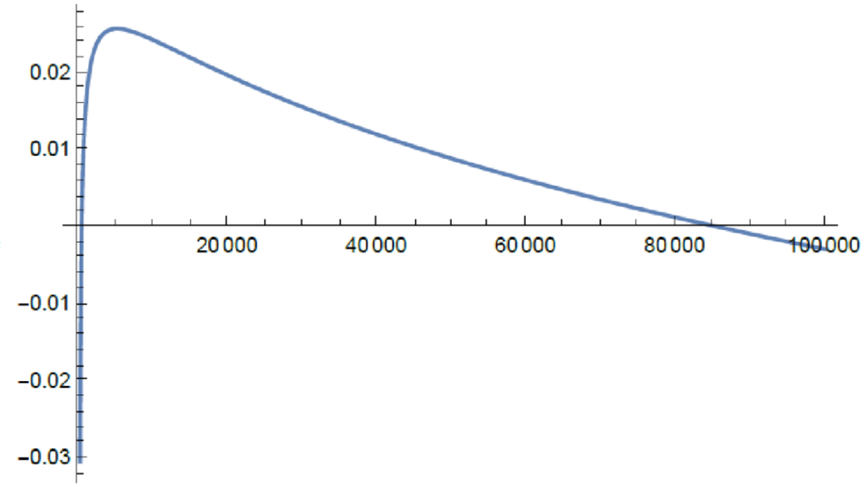

SM Figure 1. Fitted relationship (equation (SM 2)) between real annual per capita GDP (x-axis) and growth in real annual per capita GDP (y-axis).

In the *Accelerated development in low-income countries* and *Equitable development* scenarios, we used an accelerated growth equation for countries with per capita GDP less than $8,100:

$$\frac{d(per\ capita\ GDP(t))}{dt} = per\ capita\ GDP(t) \times [0.16 - 0.015 \times \ln(per\ capita\ GDP(t))\ ] \quad \text{(SM 5)}$$

Once a country's real annual per capita GDP reaches $8,100, its real annual per capita GDP growth trajectory reverts to the growth equation shown in equation (SM 4). Equation (SM 5) translates to an approximate maximum of 4.5% per annum growth rate for per capita GDP. This growth rate was observed in the low-middle income country group from 1988-2018. The low-middle income country group was the fastest growing group from 1998-2018.

See SM Table 3 for 2018, 2050, and 2100 GDP per capita projections for each country under each scenario.

SM Table 3: Per capita GDP by country (2010 constant $)

Country		BAU and Reduced per capita crop demand in high-income countries		Accelerated development in low-income countries and Equitable development	
	2018	2050	2100	2050	2100
Afghanistan	564	853	2322	2940	12625
Albania	5047	11273	30396	11773	31264
Algeria	4816	10795	29546	11379	30583
Angola	3230	7335	22584	8669	25456
Argentina	10070	20426	43512	20426	43512
Armenia	4407	9934	27953	10682	29340
Australia	57135	67952	78071	67952	78071
Austria	49999	62706	75439	62706	75439
Azerbaijan	5765	12720	32844	13005	33305
Bahrain	21438	36046	58533	36046	58533
Bangladesh	1203	2418	8589	4699	16779
Belarus	6768	14653	35851	14739	35980
Belgium	46439	59932	73976	59932	73976
Belize	4310	9727	27558	10517	29040
Benin	896	1651	5630	3917	14971
Bhutan	3173	7205	22288	8571	25253
Bolivia	2560	5769	18818	7497	22966
Bosnia and Herzegovina	6056	13291	33762	13506	34101
Botswana	8031	16958	39119	16958	39119
Brazil	11026	21959	45292	21959	45292
Brunei Darussalam	31437	46753	66203	46753	66203
Bulgaria	8617	17985	40478	17985	40478
Burkina Faso	710	1195	3755	3390	13721
Burundi	211	156	44	1599	9104
Cambodia	1205	2424	8610	4704	16791
Cameroon	1498	3161	11195	5381	18320
Canada	51336	63719	75960	63719	75960
Central African Republic	385	466	810	2321	11060
Chad	813	1446	4798	3688	14432
Chile	15130	28006	51562	28006	51562
China	7401	15824	37551	15845	37580
Colombia	7696	16360	38301	16366	38309
Congo Dem. Rep.	419	535	1059	2446	11382
Congo Rep.	2652	5988	19375	7663	23324
Costa Rica	9893	20135	43164	20135	43164
Cote d'Ivoire	1693	3649	12787	5804	19263

Country		BAU and Reduced per capita crop demand in high-income countries		Accelerated development in low-income countries and Equitable development	
	2018	2050	2100	2050	2100
Croatia	15614	28671	52191	28671	52191
Cuba	6665	14459	35563	14561	35715
Cyprus	22598	37397	59588	37397	59588
Czech Republic	23257	38151	60165	38151	60165
Denmark	63384	72250	80110	72250	80110
Djibouti					
Dominican Republic	7751	16458	38436	16462	38441
Ecuador	5185	11556	30890	12011	31667
Egypt Arab Rep.	2907	6590	20851	8112	24289
El Salvador	3511	7974	23994	9153	26435
Equatorial Guinea	10602	21285	44521	21285	44521
Eritrea					
Estonia	19918	34220	57059	34220	57059
Eswatini	4820	10803	29561	11386	30595
Ethiopia	570	868	2384	2961	12677
Finland	48540	61583	74853	61583	74853
France	45005	58782	73354	58782	73354
Gabon	9077	18773	41486	18773	41486
Gambia The	519	752	1905	2793	12259
Georgia	4166	9417	26956	10271	28586
Germany	47390	60684	74378	60684	74378
Ghana	1807	3934	13678	6044	19794
Greece	24018	39009	60811	39009	60811
Guatemala	3160	7176	22221	8549	25208
Guinea	943	1767	6098	4043	15266
Guinea-Bissau	622	988	2884	3124	13078
Guyana	3966	8983	26094	9930	27945
Haiti	730	1245	3961	3450	13865
Honduras	2204	4912	16530	6834	21528
Hungary	16608	30007	53422	30007	53422
Iceland	54712	66215	77217	66215	77217
India	2104	4668	15845	6641	21104
Indonesia	4285	9673	27453	10473	28959
Iran Islamic Rep.	6857	14819	36098	14894	36209
Iraq	5511	12214	32010	12568	32596
Ireland	79334	82216	84489	82216	84489
Israel	36873	51850	69389	51850	69389
Italy	35277	50397	68506	50397	68506
Jamaica	4842	10849	29643	11424	30661
Japan	48661	61676	74902	61676	74902
Jordan	3234	7345	22606	8676	25471
Kazakhstan	11139	22136	45492	22136	45492
Kenya	1202	2416	8581	4697	16774
Korea Dem. People's Rep.					
Korea Rep.	27004	42252	63160	42252	63160
Kuwait	33538	48773	67496	48773	67496
Kyrgyz Republic	1089	2132	7518	4419	16136
Lao PDR	1789	3890	13540	6007	19712

Country		BAU and Reduced per capita crop demand in high-income countries		Accelerated development in low-income countries and Equitable development	
	2018	2050	2100	2050	2100
Latvia	16389	29717	53158	29717	53158
Lebanon	6240	13648	34322	13824	34595
Liberia	541	802	2111	2867	12444
Libya	7529	16058	37879	16071	37898
Lithuania	17596	31301	54577	31301	54577
Madagascar	433	564	1167	2496	11509
Malawi	517	747	1886	2786	12242
Malaysia	12110	23634	47140	23634	47140
Mali	778	1360	4440	3588	14193
Mauritania	1349	2787	9913	5045	17563
Mexico	10385	20937	44115	20937	44115
Moldova	2349	5263	17490	7109	22125
Mongolia	4198	9486	27091	10326	28687
Montenegro	8155	17177	39413	17177	39413
Morocco	3410	7746	23498	8979	26087
Mozambique	539	797	2092	2860	12427
Myanmar	1572	3347	11813	5545	18685
Namibia	6073	13324	33814	13535	34146
Nepal	812	1443	4785	3684	14423
Netherlands	55594	66852	77533	66852	77533
New Zealand	39141	53859	70579	53859	70579
Nicaragua	1860	4067	14082	6154	20037
Niger	399	494	908	2372	11191
Nigeria	2396	5378	17796	7197	22316
Norway	91714	89161	87295	89161	87295
Oman	15664	28738	52254	28738	52254
Pakistan	1197	2402	8529	4683	16743
Panama	11724	23045	46501	23045	46501
Papua New Guinea	2400	5387	17821	7205	22332
Paraguay	5394	11981	31617	12368	32266
Peru	6454	14058	34955	14193	35161
Philippines	3022	6857	21483	8311	24710
Poland	16665	30083	53490	30083	53490
Portugal	23797	38761	60625	38761	60625
Qatar	63222	72142	80060	72142	80060
Romania	11516	22724	46148	22724	46148
Russian Federation	11817	23188	46658	23188	46658
Rwanda	826	1478	4929	3724	14517
Saudi Arabia	20773	35255	57901	35255	57901
Senegal	1547	3283	11603	5489	18561
Serbia	5457	12107	31830	12477	32446
Sierra Leone	474	653	1510	2641	11879
Slovak Republic	20647	35103	57779	35103	57779
Slovenia	26624	41849	62876	41849	62876
Somalia					
South Africa	7431	15880	37629	15899	37657
Spain	33168	48423	67275	48423	67275
Sri Lanka	4018	9097	26323	10019	28115
Sudan	1856	4055	14046	6144	20015
Suriname	8049	16989	39160	16989	39160

Country		BAU and Reduced per capita crop demand in high-income countries		Accelerated development in low-income countries and Equitable development	
	2018	2050	2100	2050	2100
Sweden	58446	68876	78517	68876	78517
Switzerland	78732	81863	84342	81863	84342
Syrian Arab Republic					
Tajikistan	1073	2091	7363	4378	16043
Tanzania	929	1732	5957	4005	15177
Thailand	6362	13881	34684	14034	34919
Togo	672	1107	3382	3279	13453
Tunisia	4402	9924	27934	10674	29326
Turkey	15023	27858	51421	27858	51421
Turkmenistan	7648	16273	38180	16281	38191
Uganda	710	1195	3753	3389	13720
Ukraine	2971	6739	21206	8223	24525
United Arab Emirates	40782	55275	71396	55275	71396
United Kingdom	42568	56781	72248	56781	72248
United States	54554	66099	77160	66099	77160
Uruguay	14618	27292	50876	27292	50876
Uzbekistan	2056	4551	15510	6548	20901
Venezuela RB					
Vietnam	1964	4324	14850	6365	20500
Yemen Rep.	668	1096	3337	3265	13419
Zambia	1672	3599	12627	5761	19168
Zimbabwe	1322	2719	9674	4982	17422

To ensure that our GDP per capita projections are reasonable we compared our *BAU* and *Reduced per capita crop demand in high-income country* scenario projections in 2050 and 2100 to other published projections. First, we considered GDP projections in Fontagné et al. (2022).[96] In SM Figure 2 the projections of Fontagné et al. (2022) are on the horizontal axes and our *BAU* and *Reduced per capita crop demand in high-income country* scenario projections are on the vertical axes. The correlation between our country-level projections and the Fontagné et al. (2022) estimates are quite high, especially for 2050. Our estimates for growth tend to be slightly more conservative at the upper end than those of Fontagné et al. (2022).

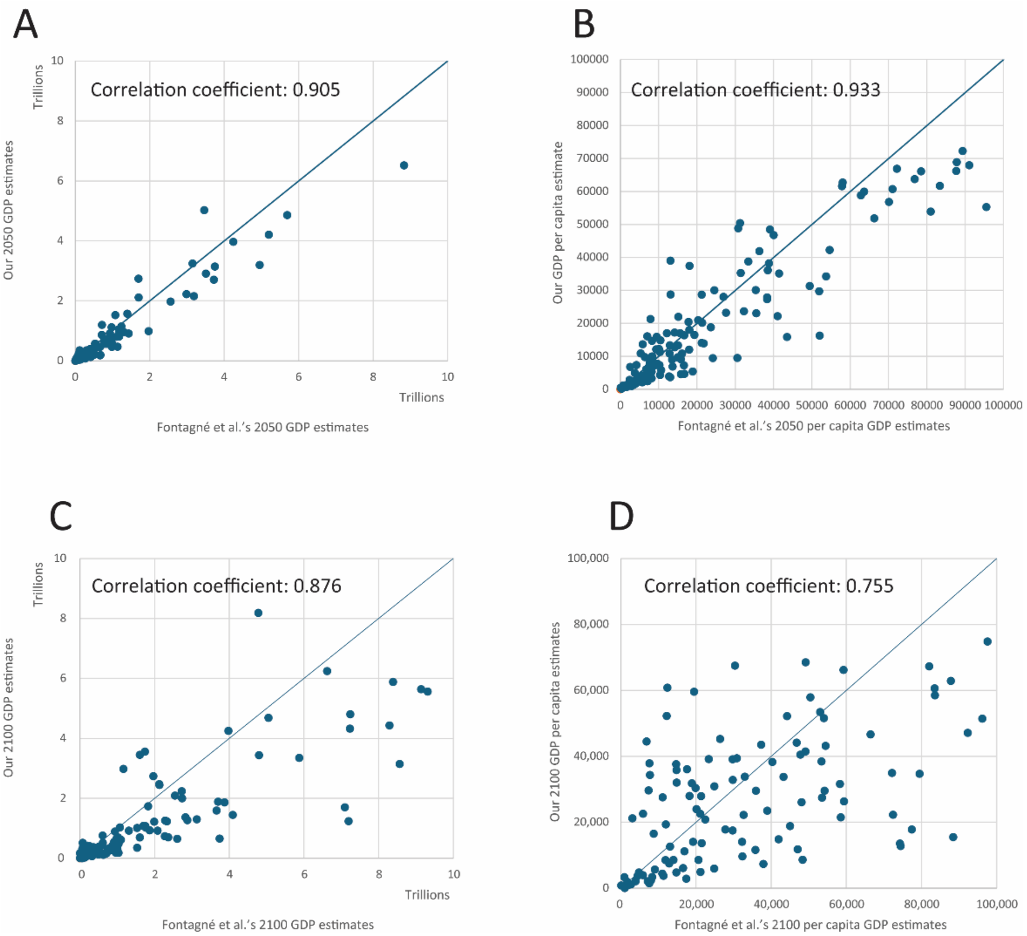

SM Figure 2. The estimates of Fontagné et al. (2022) are on the horizontal axes and our estimates are on the vertical axes. GDP and GDP per capita in 2050 (A and B, respectively). GDP and GDP per capita in 2100 (C and D, respectively).

Next, we considered two sets of GDP projections used in Rennert, K., et al. (2022).[97] The first set of comparative GDP projections are what Rennert et al. (2022) labeled IIASA GDP_SSP2. SSP2 refers to the IPCC's Shared Socioeconomic Pathways second scenario, known as "Middle of the Road (Medium challenges to mitigation and adaptation)" These country-level 2050 and 2100 IIASA GDP estimates were found at https://tntcat.iiasa.ac.at/SspDb/dsd?Action=htmlpage&page=10. In SM Figure 3 the IIASA GDP_SSP2 projections are on the horizontal axes and our *BAU* and *Reduced per capita crop demand in high-income country* scenario projections are on the vertical axes. Once again, the correlation between the projections is quite high. Our estimates for growth tend to be slightly more conservative than the projections by IIASA.

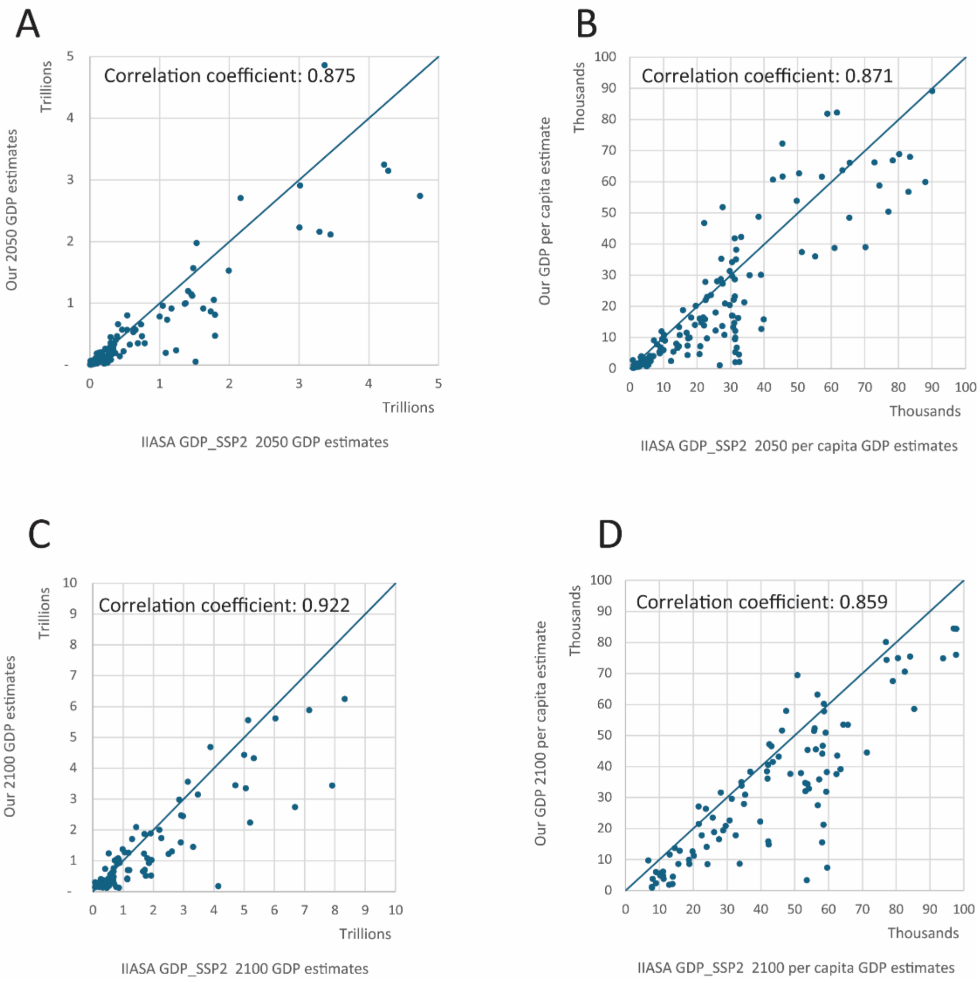

SM Figure 3. IIASA GDP_SSP2 estimates are on the horizontal axes and our BAU and LC scenario estimates are on the vertical axes. GDP and GDP per capita in 2050 (A and B, respectively). GDP and GDP per capita in 2100 (C and D, respectively).

The last set of comparative GDP projections come from what Rennert et al. (2022) labeled OECD Env-Growth_SSP2. The OECD Env-Growth GDP estimates are also found at the IIASA Database (https://tntcat.iiasa.ac.at/SspDb/dsd?Action=htmlpage&page=10). Here again, the SSP2 refers to the "Middle of the Road (Medium challenges to mitigation and adaptation)" path. In SM Figure 4 the OECD Env-Growth GDP estimates are on the horizontal axes and our *BAU* and *Reduced per capita crop demand in high-income country* scenario projections are on the vertical axes. Our estimates for growth in GDP and GDP per capita tend to be somewhat more conservative than the OECD estimates especially for 2100. Faster growth in income, especially for high-income countries, would tend to reduce cropland area needs in the future (as discussed above).

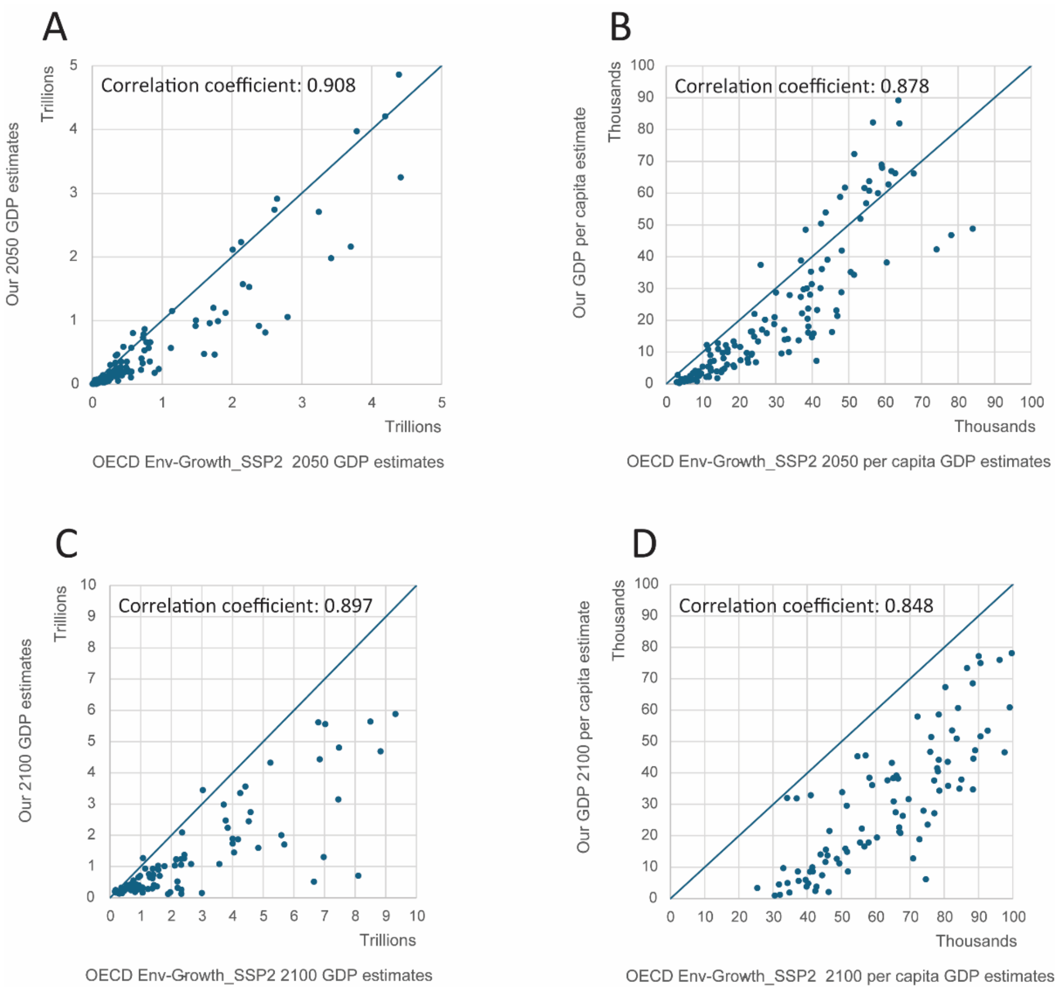

SM Figure 4. OECD Env-Growth GDP estimates are on the horizontal axes and our BAU and LC scenario estimates are on the vertical axes. GDP and GDP per capita in 2050 (A and B, respectively). GDP and GDP per capita in 2100 (C and D, respectively).

To convert a country's real GDP per capita into its daily kcal crop demand per capita we estimated the relationship between real per capita GDP (2010 USD) and per capita daily kcal crop demand at the income group-level for 2014-2018. Specifically, we plotted the mean values for real annual per capita GDP against per capita daily kcal crop demand for each income group (low; escaped low; lower middle; escaped lower middle; upper middle, escaped upper middle, and high; China; and India) for each year between 2014 and 2018. The curve that best fit this plot is plotted in SM Figure 5 and has the form,

$$\ln daily\ kcal\ crop\ demand\ capita^{-1}(t) = 6.431988 + \left[0.240283 \times \ln\left(GDP\ cap^{-1}\ (t)\right)\right]. \quad \text{(SM 6)}$$

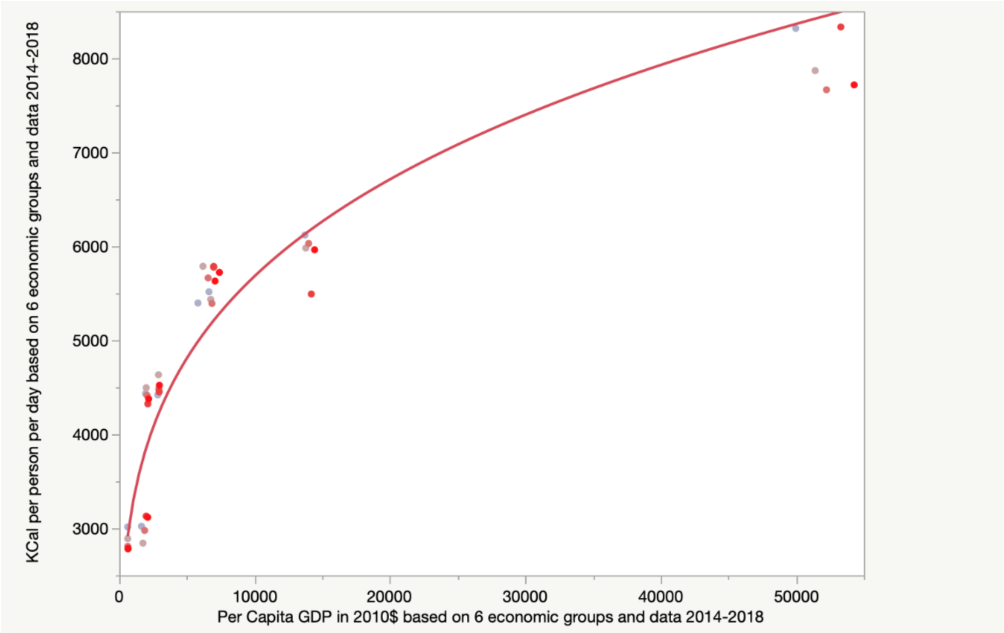

SM Figure 5. Fitted relationship between real annual per capita GDP at the country-group level (x-axis) and daily kcal crop demand per capita at the country group level (y-axis).

We then used the observed country-level annual per capita GDP from 2018 and projected annual per capita GDP from 2050 and 2100 (generated with equations (SM 4) and (SM 5)) into equation (SM 6) to produce predicted 2018, 2050, and 2100 per capita daily kcal crop demand for each country. Because country-level per capita GDP trajectories differ by scenario, we created one set of initial predicted country-level daily crop demand levels for the *BAU* and *Reduced per capita crop demand in high-income countries* scenarios and another set of initial predicted country-level daily crop demand levels for the *Accelerated development in low-income countries* and *Equitable development* scenarios. Next, using the per capita daily kcal crop demand adjustment rules detailed in SM Table 1 we generated final 2050 and 2100 per capita daily kcal crop demand for each country in each economic scenario. (SM Table 4).

SM Table 4: Per capita kcal crop demand by country and scenario

		BAU		***Accelerated development in low-income countries***		***Reduced crop demand in high-income countries***		***Equitable development***	
**Country**	**2018**	**2050**	**2100**	**2050**	**2100**	**2050**	**2100**	**2050**	**2100**
Afghanistan	1245	1543	2398	3816	5000	1543	2398	3816	5000
Angola	2293	3236	4873	4226	5074	3236	4873	4226	5000
Albania	4255	5282	6856	5343	6906	5141	5000	5172	5000
Argentina	24810	25865	27209	25865	27209	15432	5000	15432	5000
Armenia	2810	3817	5418	4458	5503	3817	5000	4458	5000
Australia	12096	12463	12769	12463	12769	8732	5000	8732	5000
Austria	8437	8905	9306	8905	9306	6952	5000	6952	5000
Azerbaijan	4293	5335	6876	5367	6902	5168	5000	5184	5000
Burundi	1953	1797	1248	4181	5260	1797	1248	4181	5000
Belgium	15165	15684	16138	15684	16138	10342	5000	10342	5000

		BAU		*Accelerated development in low-income countries*		*Reduced crop demand in high-income countries*		*Equitable development*	
**Country**	**2018**	**2050**	**2100**	**2050**	**2100**	**2050**	**2100**	**2050**	**2100**
Benin	5387	5890	7154	6741	8465	5445	5000	5870	5000
Burkina Faso	3424	3825	4905	4898	6545	3825	4905	4898	5000
Bangladesh	2748	3372	4811	4536	5767	3372	4811	4536	5000
Bulgaria	12463	13523	14931	13523	14931	9262	5000	9262	5000
Belarus	10919	11974	13468	11983	13475	8487	5000	8492	5000
Belize	9291	10294	11899	10401	11991	7647	5000	7700	5000
Bolivia	8923	9806	11442	10129	11766	7403	5000	7565	5000
Brazil	15423	16470	17774	16470	17774	10735	5000	10735	5000
Brunei Darussalam	251	1000	1717	3000	5000	1000	1717	3000	5000
Bhutan	2816	3756	5393	4490	5603	3756	5000	4490	5000
Botswana	1409	2469	3904	3735	5000	2469	3904	3735	5000
Central African Republic	2505	2627	3014	4454	5729	2627	3014	4454	5000
Canada	26437	26886	27268	26886	27268	15943	5000	15943	5000
Switzerland	2871	2959	3026	3979	5000	2959	3026	3979	5000
Chile	4649	5649	6799	5649	6799	5325	5000	5325	5000
China	5722	6781	8245	6783	8246	5890	5000	5891	5000
Cote d'Ivoire	3855	4607	6175	5133	6799	4607	5000	5067	5000
Cameroon	4962	5670	7200	6257	7933	5335	5000	5629	5000
Congo Dem. Rep.	1840	2001	2502	4120	5051	2001	2502	4120	5000
Congo Rep.	1577	2470	4107	3888	5000	2470	4107	3888	5000
Colombia	4741	5801	7252	5802	7252	5401	5000	5401	5000
Costa Rica	6517	7572	8924	7572	8924	6286	5000	6286	5000
Cuba	3984	5038	6537	5049	6545	5019	5000	5024	5000
Czech Republic	10935	11814	12721	11814	12721	8407	5000	8407	5000
Germany	9453	9958	10397	9958	10397	7479	5000	7479	5000
Denmark	15231	15514	15744	15514	15744	10257	5000	10257	5000
Dominican Republic	3710	4770	6218	4885	6218	4770	5000	4885	5000
Algeria	4804	5825	7409	5898	7470	5412	5000	5449	5000
Ecuador	4086	5117	6685	5172	6729	5058	5000	5086	5000
Egypt Arab Rep.	4534	5452	7091	5715	7344	5226	5000	5357	5000
Spain	12313	13035	13717	13035	13717	9017	5000	9017	5000
Estonia	6457	7388	8386	7388	8386	6194	5000	6194	5000
Ethiopia	2657	2960	3828	4522	5817	2960	3828	4522	5000
Finland	4571	5060	5482	5060	5482	5030	5000	5030	5000
France	10532	11072	11548	11072	11548	8036	5000	8036	5000
Gabon	1757	2816	4204	3908	5000	2816	4204	3908	5000
United Kingdom	4852	5429	5943	5429	5943	5215	5000	5215	5000
Georgia	2483	3480	5090	4299	5192	3480	5000	4299	5000
Ghana	5932	6707	8292	7200	8861	5853	5000	6100	5000
Guinea	5028	5553	6852	6377	8096	5276	5000	5689	5000
Gambia The	1993	2253	3017	4192	5169	2253	3017	4192	5000
Guinea-Bissau	2235	2578	3534	4308	5380	2578	3534	4308	5000
Equatorial Guinea	937	1988	3309	3494	5000	1988	3309	3494	5000
Greece	9185	10052	10939	10052	10939	7526	5000	7526	5000

		BAU		Accelerated development in low-income countries		Reduced crop demand in high-income countries		Equitable development	
**Country**	**2018**	**2050**	**2100**	**2050**	**2100**	**2050**	**2100**	**2050**	**2100**
Guatemala	6159	7097	8735	7323	8946	6049	5000	6161	5000
Guyana	11141	12129	13746	12264	13865	8564	5000	8632	5000
Honduras	5169	6008	7629	6403	8049	5504	5000	5702	5000
Haiti	1371	1785	2890	3871	5000	1785	2890	3871	5000
Hungary	17458	18438	19538	18438	19538	11719	5000	11719	5000
Indonesia	8346	9348	10954	9457	11047	7174	5000	7228	5000
India	3117	3942	5557	4680	6009	3942	5000	4680	5000
Ireland	4886	4967	5029	4983	5029	4967	5000	4983	5000
Iran Islamic Rep.	4686	5742	7231	5749	7237	5371	5000	5375	5000
Iraq	1667	2705	4258	3873	5000	2705	4258	3873	5000
Israel	4442	5106	5718	5106	5718	5053	5000	5053	5000
Italy	6051	6740	7381	6740	7381	5870	5000	5870	5000
Jordan	2508	3452	5088	4334	5290	3452	5000	4334	5000
Japan	3056	3543	3964	4272	5000	3543	3964	4272	5000
Kazakhstan	10028	11074	12374	11074	12374	8037	5000	8037	5000
Kenya	1854	2478	3915	4089	5000	2478	3915	4089	5000
Kyrgyz Republic	3948	4532	5918	5282	6987	4532	5000	5141	5000
Cambodia	5257	5881	7321	6580	8275	5441	5000	5790	5000
Korea Rep.	3317	4136	4951	4568	5000	4136	4951	4568	5000
Kuwait	2488	3204	3879	4102	5000	3204	3879	4102	5000
Lao PDR	5234	6005	7588	6504	8165	5503	5000	5752	5000
Lebanon	2443	3492	5011	4256	5026	3492	5000	4256	5000
Liberia	1364	1643	2454	3876	5000	1643	2454	3876	5000
Libya	4673	5733	7191	5734	7192	5366	5000	5367	5000
Sri Lanka	2889	3879	5495	4505	5609	3879	5000	4505	5000
Lithuania	11924	12890	13958	12890	13958	8945	5000	8945	5000
Latvia	9342	10325	11432	10325	11432	7663	5000	7663	5000
Morocco	5266	6222	7855	6415	8033	5611	5000	5708	5000
Moldova	9797	10656	12285	11021	12662	7828	5000	8010	5000
Madagascar	1532	1709	2252	3966	5000	1709	2252	3966	5000
Mexico	6097	7149	8480	7149	8480	6074	5000	6074	5000
Mali	5468	5910	7067	6833	8572	5455	5000	5917	5000
Myanmar	5454	6180	7726	6743	8415	5590	5000	5872	5000
Mongolia	1848	2846	4455	3981	5000	2846	4455	3981	5000
Mozambique	2246	2524	3331	4318	5416	2524	3331	4318	5000
Mauritania	1934	2602	4092	4121	5000	2602	4092	4121	5000
Malawi	4557	4815	5575	5949	7734	4815	5000	5474	5000
Malaysia	33693	34730	35991	34730	35991	19865	5000	19865	5000
Namibia	1767	2814	4341	3919	5000	2814	4341	3919	5000
Niger	4294	4432	4867	5696	7512	4432	4867	5348	5000
Nigeria	4305	5169	6800	5524	7165	5085	5000	5262	5000
Nicaragua	3989	4773	6365	5252	6911	4773	5000	5126	5000
Netherlands	9982	10371	10696	10371	10696	7686	5000	7686	5000
Norway	2211	2145	2096	3573	5000	2145	2096	3573	5000
Nepal	3518	3978	5169	4940	6615	3978	5000	4940	5000
New Zealand	3966	4594	5166	4797	5166	4594	5000	4797	5000
Oman	2307	3301	4432	4150	5000	3301	4432	4150	5000
Pakistan	2451	3073	4508	4387	5471	3073	4508	4387	5000
Panama	2884	3925	5201	4462	5201	3925	5000	4462	5000

		BAU		*Accelerated development in low-income countries*		*Reduced crop demand in high-income countries*		*Equitable development*	
**Country**	**2018**	**2050**	**2100**	**2050**	**2100**	**2050**	**2100**	**2050**	**2100**
Peru	4682	5734	7242	5748	7253	5367	5000	5374	5000
Philippines	3851	4778	6417	5023	6650	4778	5000	5012	5000
Papua New Guinea	6426	7291	8922	7645	9285	6145	5000	6323	5000
Poland	8736	9716	10814	9716	10814	7358	5000	7358	5000
Korea Dem. People's Rep.	1897	3212	4057	4640	6052	3212	4057	4640	5000
Portugal	6140	7010	7903	7010	7903	6005	5000	6005	5000
Paraguay	30031	31066	32625	31112	32661	18033	5000	18056	5000
Romania	14878	15921	17205	15921	17205	10460	5000	10460	5000
Russian Federation	7590	8630	9902	8630	9902	6815	5000	6815	5000
Rwanda	2001	2469	3673	4181	5094	2469	3673	4181	5000
Saudi Arabia	3258	4175	5149	4588	5149	4175	5000	4588	5000
Sudan	4873	5656	7247	6137	7795	5328	5000	5568	5000
Senegal	2487	3206	4747	4389	5451	3206	4747	4389	5000
Sierra Leone	1385	1604	2261	3890	5000	1604	2261	3890	5000
El Salvador	3978	4941	6572	5122	6737	4941	5000	5061	5000
Suriname	3408	4469	5903	4734	5903	4469	5000	4734	5000
Slovak Republic	8736	9656	10633	9656	10633	7328	5000	7328	5000
Slovenia	3268	4093	4917	4547	5000	4093	4917	4547	5000
Sweden	4173	4523	4812	4761	5000	4523	4812	4761	5000
Eswatini	7999	9020	10604	9094	10665	7010	5000	7047	5000
Syrian Arab Republic	2003	5105	6725	5107	6735	5052	5000	5053	5000
Chad	3697	4158	5351	5059	6793	4158	5000	5029	5000
Togo	2825	3202	4234	4600	5956	3202	4234	4600	5000
Thailand	9267	10318	11831	10334	11844	7659	5000	7667	5000
Tajikistan	2966	3544	4922	4651	6008	3544	4922	4651	5000
Turkmenistan	3215	4275	5727	4638	5728	4275	5000	4638	5000
Tunisia	4806	5812	7413	5912	7499	5406	5000	5456	5000
Turkey	7382	8384	9537	8384	9537	6692	5000	6692	5000
Tanzania	3666	4184	5472	5016	6737	4184	5000	5008	5000
Uganda	1935	2336	3416	4154	5057	2336	3416	4154	5000
Ukraine	17140	18063	19702	18316	19944	11532	5000	11658	5000
Uruguay	13176	14184	15351	14184	15351	9592	5000	9592	5000
United States	16908	17311	17650	17311	17650	11155	5000	11155	5000
Uzbekistan	4923	5740	7352	6170	7821	5370	5000	5585	5000
Venezuela RB	1877	6723	7968	6736	7975	5861	5000	5868	5000
Vietnam	4843	5645	7248	6097	7751	5322	5000	5549	5000
Yemen Rep.	1374	1748	2773	3875	5000	1748	2773	3875	5000
South Africa	3847	4906	6369	4954	6370	4906	5000	4954	5000
Zambia	2398	3146	4711	4339	5345	3146	4711	4339	5000
Zimbabwe	1390	2051	3532	3851	5000	2051	3532	3851	5000

c. Yield
We used data from UN FAO [main text ref. 5], USDA NASS [main text ref. 88], and Brazil's Instituto Brasileiro de Geografia e Estatística [main text ref. 89] and the methods of Ray et al. (2012, 2013, 2019) [main text ref. 90-92] to compile a 1980 to 2018 panel of administrative unit-level yields and harvested hectares for 23 focus crops (SM Table 5). These 23 crops supplied the vast majority of produced kcals. As of 2018 the top ten crops comprised 83% of global food calories and 63% of harvested cropland [main text ref 92]. This dataset is normalized so that it agrees with FAO data at the national level.

SM Table 5. The 23 crops used in our future yield calculations

Apple	Oats	Sorghum
Barley	Oil palm	Soybean
Bean	Orange	Sugarbeet
Cassava	Potato	Sugarcane
Cotton	Rapeseed	Sunflower
Grape	Rice	Sweet potato
Maize	Rye	Tomato
Millet		Wheat

We estimated country-level yields in kcals per ha for the years 2050 and 2100 under the four different scenarios listed in SM Table 1. For each crop-administrative unit combination (a country can be made up of one or more administrative units), we projected yield into the future via linear extrapolation of historical yield data set described above, subject to the constraint that future yield does not exceed the administrative unit's yield ceiling for that crop. We determined crop-administrative unit yield ceilings from a quantile regression model of yields following the methods in Gerber et al. (2024) [main text ref. 93], with modifications described in SM Table 6.

SM Table 6. Modifications to Gerber et al. (2024) attainable yield model

**Description of modifications to Gerber et al. (2024) attainable yield model**	**Notes**
Addition of Extended Degree Day (EDD) term.	Provides means for model to capture response to extreme weather.[98]
Range of base values for EDD term.	Allows for different crops to have different thresholds of critical temperature. (We use the Meinshausen loss criterion to assess best-fit critical temperature threshold.[99])
Addition of cross terms between EDD and Irrigation; GDD terms.	Capture interactions between extreme heat and increased irrigation requirements; increase in EDD and GDD.
Vernalization factor (VF) removed from model.	VF is discontinuous, hence inappropriate in this model which uses space-for-time substitution.
Cross-validation in region (in addition to time).	Allows for assessment of values across regions (in contrast to Gerber et al. (2024), which focused analysis on relative time trends).
Add 0 labels where there is no crop.	Follow methods of Guilpart et al. (2022)[100], helps to bound regional cross-validation.

We calculated administrative unit-level yield ceilings for each year from the historical yield data, and then future yield ceilings are extrapolated from individual-year model results. Yield ceilings are not allowed to grow past 2050 to inhibit unbounded growth. These methods for calculating

future yield ceiling closely follow those used in the World Bank *Changing Wealth of Nations* 2021[101]. All calculations are carried out at the level of the administrative unit and aggregated to the country level via an average weighted by year 2018 harvested areas. An example of the yield calculation results for a single crop-country combination (maize-Argentina) is shown in SM Figure 6.

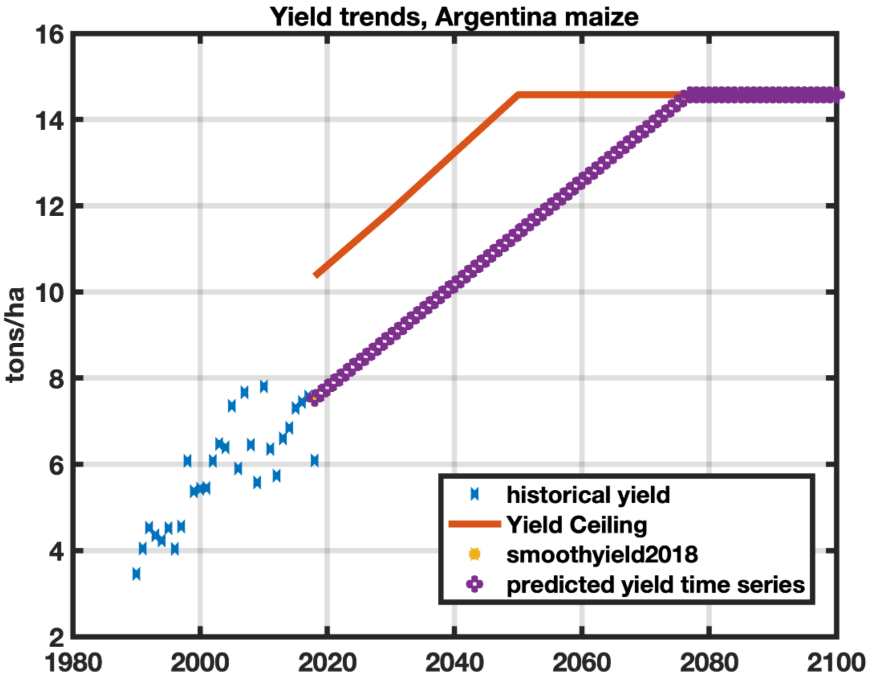

SM Figure 6. Example of yield calculation results for maize in Argentina. Years on x-axis and Mg ha^{-1} on the y-axis. Here the impacts of climate change are not considered.

The dataset we used to project yields and calculate yield ceilings was based on crop yield and area data from Ray et al. (2019) [main text ref. 92] with some modification and augmentation as described in SM Table 7.

SM Table 7. Modifications to Ray et al. (2019) crop yield and area dataset

**Description of modifications to Ray et al. (2019) crop yield and area dataset**	**Notes**
Normalize country-level yield and area to FAO data.	March 2023 version of FAO data [main text ref. 5].
Extend data to years 2013-2021.	Data extended using a perturbation approach. For each country c, define Y_{avg}^{i} as the average crop yield within each political unit i over 2008-2012, then determine yield in year 20xx as $Y_{avg}^{i}\frac{Y_{FAO}^{20xx}}{Y_{FAO}^{2010}}$ with an analogous procedure for determining area.
Extend data for non-Ray et al. (2019) crops to years 2000-2021.	Data extended using a perturbation approach. For each country c, define Y_{avg}^{i} as the crop yield in the Earthstat dataset (Monfreda, et al. 2008)[102], then determine yield in 20xx as $Y_{avg}^{i}\frac{Y_{FAO}^{20xx}}{Y_{FAO}^{2000}}$ with an analogous procedure for determining area.

Description of modifications to Ray et al. (2019) crop yield and area dataset	Notes
Replace data for the period 2013-2021 for USA and Brazil with data from their national agricultural statistics agencies (main text refs. 88 and 89).	These two datasets represent approximately 46% of the total number of administrative units for which there is data and provide substantial coverage in climate space.

c.1. Details on the model used to project yields to the years 2050 and 2100

The administrative unit-level crop yield model uses a set of two temperature variables, two precipitation variables, three soil variables, and one topography variable, all defined at the administrative unit-level, to estimate a crop's annual yield in year *t* for administrative unit *n*.

The two temperature terms are growing degree days (GDD) and extreme degree days (EDD). GDD is a commonly used agronomic term that estimates crop development, calculated as the sum of daily temperature values over a threshold (or "base) temperature Tb. In contrast, EDD is a measure of extreme temperatures that are detrimental to yield and is a sum of daily temperatures above a threshold (or "critical") temperature Tc. Here, we determine Tb from the literature, and we determine Tc empirically. Like temperature, precipitation is included through both mean annual precipitation (MAP) and an estimate of its distribution over the growing season (Precipitation Concentration Index (PCI)). We include the interaction of mean annual precipitation with all terms as water availability may alleviate stress from excess temperature, mild temperatures are of little benefit to a moisture-starved crop, and more concentrated precipitation may provide less usable water.

Two soil variables are included in the model as well: organic carbon content (SOC) and pH (PH). SOC and pH are both proxies for soil fertility. SOC helps retain nutrients and pH affects whether the nutrients are available for the plant to access. We hold each administrative unit's SOC and PH levels constant.

Irrigation is included as a management variable. It is also interacted with all climatic terms as it has a similar influence on yield as mean annual precipitation. We describe the explanatory variables in SM Table 8.

The functional form of the yield model for crop *j* in administrative unit *n* in year *t* is:

$$Y_{jtn} = \alpha_j + \beta_{1jt}GDD_{tn} + \beta_{2jt}GDD_{tn}^2 + \beta_{3jt}EDD_{tnc_j} + \beta_{4jt}MAP_{tn} +$$

$$\beta_{5jt}MAP_{tn}^2 + \beta_{6jt}PCI_{tn} + \beta_{7jt}(GDD_{tn} \times MAP_{tn}) +$$

$$\beta_{8jt}\left(EDD_{tnc_j} \times MAP_{tn}\right) + \beta_{9jt}(PCI_{tn} \times MAP_{tn}) + \beta_{10jt}IRR_{tn} +$$

$$\beta_{11jt}(GDD_{tn} \times IRR_{tn}) + \beta_{12jt}(IRR_{tn} \times EDD_{tnc_j}) +$$

$$\beta_{13jt}(IRR_{tn} \times MAP_{tn}) + \beta_{14jt}(IRR_{tn} \times PCI_{tn}) +$$

$$\beta_{15jt}SOC_n + \beta_{16jt}PH_n \quad \text{(SM 7)}$$

We use a model selection process following that of Gerber et al (2024). We start with a model with all the terms as given in (SM 7) and use a cross-validation process to remove terms leading to the lowest possible modified Meinshausen error criterion (equation 1 from Meinshausen et al. (2006) modified following Gerber et al. (2024) [main text ref. 93]). The terms which remain after this process for each crop are shown in SM Table 9.

c.2. Data used to project yields to the years 2050 and 2100

Using country-level data from UN FAO [main text ref. 5] and the methods of Ray et al. (2012) [main text ref. 90] and Ray et al. (2013) [main text ref. 91], we compiled tables of annual yield and harvested area data for ten crops (maize, wheat, rice, soy, barley, cassava, oil palm, rapeseed, sorghum, sugarcane) for 1973 to 2018 at the country-administrative unit level. A data quality check for each crop-year combination rejected observations from administrative units with harvested area greater than 300% (i.e. multi-cropping index for the entire region exceeds 3) or yield values greater than 2 standard deviations above the area-weighted 97.5th percentile yield value.

We used an analogous procedure to extend the Earthstat dataset (Monfreda, et al. 2008) from 2000 to generate tables of annual yield and harvested area data at the country- administrative level from 1980 to 2018 for an additional 13 crops. Therefore, we have a 1980 to 2018 panel of country-administrative unit-level yields and harvested hectares for 23 focus crops (SM Table 5).

An exception to this general procedure for determining the panel of yields and harvested hectares was made for the US and Brazil. Harvested yield and area data from the National Agricultural Statistics Service of the USA (https://www.nass.usda.gov) (downloaded December 2023; main text ref. 88) and the Brazilian Institute of Geography and Statistics (https://www.ibge.gov.br/) (downloaded January 2024; main text ref. 89) were used in lieu of the above for the following crops:

- USA: barley, cotton, maize, oats, potato, rapeseed, rice, rye, sorghum, soybean, sugarcane, sweet potato, and wheat.

- Brazil: barley, cassava, cotton, maize, oats, potato, rice, rye, sorghum, soybean, sugarcane, sweet potato, tomato, and wheat.

The rationale for adding these crops is that the US and Brazil together provide about half of the independently reporting administrative units and cover substantial portions of climate space.

c.3. Biophysical data used to generate values of variables used in equation (SM 7)

<u>Climate data</u>

We reprocessed global datasets of monthly average temperature and monthly precipitation at 5 arc-minute resolution from WorldClim 2.1[103] to calculate grids of growing degree days (GDD) with base temperature (T_0) selected for each crop; mean annual precipitation (MAP); and

precipitation concentration index (PCI) (Oliver 2010)[104]. These resulting grids were incorporated into data tables as described below.

Soils data
We obtained 1-kilometer grids of available water capacity (AWCh2) soil organic carbon (ORC) and soil pH (PH) at various soil depths from the SoilGrids1km project (downloaded from www.isric.org subsequent to 2019 update; Hengl et al. 2017, FAO/IIASA/ISRIC/ISS-CAS/JRC, 2009) [105,106]. After aggregating individual depth layers to 5 arc-minute resolution, soil properties in the top 30-centimeter were obtained using a trapezoidal integration (Oliver 2010). We included topographical data by downloading 5 arc-minute grids with percentage of 100m x 100m sub-pixels with average slopes in the intervals below 10 degrees, between 10 and 30 degrees, and above 30 degrees from Harmonized World Soil Database v 1.2. (Batjes 2006).[107] These resulting grids (all at 5 arc-minute resolution) were incorporated into data tables as described below.

Extreme degree days (EDD)
We empirically determined extreme degree days (EDD) which is the cumulated sum of temperatures above a critical temperature. We constructed models for Tc of 26, 30, 34, and 38 degrees Celsius and used the model with the lowest modified Meinshausen et al. (2006) error equation (1) (following Gerber et al. (2024) [main text ref. 93]). To calculate EDD, we used the Berkeley Earth Surface Temperature data (BEST) (daily time series of maximum temperature at 1-degree resolution) in conjunction with a monthly climatology from WorldClim 2.1. We first calculated a monthly climatology from the BEST data over a time-period identical to that used to calculate the WorldClim 2.1 climatology (1970-2000) and then calculated a daily temperature anomaly relative to that monthly climatology. We then constructed daily time series of temperature anomalies relative to monthly average climatological temperatures for the entire period for which we have crop data. We thus calculated EDD on a yearly basis for each 5 arc-minute pixel.

Irrigated area
To determine a time series of irrigated area, we scaled the fraction of irrigated area as maximum proportion of crop growing area irrigated in each grid cell (IRR) from Mueller et al. (2012)[108] (which was based on MIRCA2000 (Portmann et al. 2010)[109]) using ratios of area equipped for irrigation (AEI) from Siebert et al. (2015)[110] and crop production regions from Evanson and Fuglie (2010)[111]. We use linear extrapolation of AEI to extrapolate beyond 2005, constraining the result such that IRR is between 0 and 1 (inclusive). These resulting crop-specific grids (all at 5 arc-minute resolution) were incorporated into data tables as described below.

Data tables
For each administrative unit with crop yield and area data, we used an area-weighted average (over the crop-specific harvested area) for each biophysical parameter in SM Table 8. Base growing temperatures for each crop are given in SM Table 9.

SM Table 8. Biophysical variables used in construction of the yield attainment model.

Variable	Definition	Source
GDDTb	Growing degree days, crop-specific base temperatures in administrative unit n in year t.	World Clim V2.1.
EDD_{Tc}	Extreme Degree Days (base temperature of Tc degrees C) in administrative unit n in year t.	World Clim V2.1. Berkeley Earth System Temperature.
MAP	Mean annual precipitation in administrative unit n in year t.	World Clim V2.1.
PCI	Precipitation concentration index in administrative unit n in year t.	World Clim V2.1, Hengl et al. (2017).
IRR	Fraction of area equipped for irrigation in administrative unit n in year t.	Siebert et al. (2015), Evanson and Fuglie (2010)
SOC	Soil Organic Carbon in upper 30 cm of soil in administrative unit n.	Hengl et al. 2017, FAO/IIASA/ISRIC/ISS-CAS/JRC, 2009, Batjes 2006.
PH	Soil PH in administrative unit n.	Hengl et al. 2017, FAO/IIASA/ISRIC/ISS-CAS/JRC, 2009, Batjes 2006.

SM Table 9. Base growing temperatures (degrees Celsius) and functional forms of yield model (SM 7) for each crop. (For brevity, we have not printed each variable's associated coefficient).

Crop	Base growing temp	Functional form of yield model
Apple	0	1 + GDDTb + MAP + (GDDTb x MAP) + MAP^2 + $GDDTb^2$ + PH + EDD_{30} + (GDDTb x EDD_{30})
Barley	0	1 + GDDTb + MAP + (GDDTb x MAP) + MAP^2 + $GDDTb^2$ + IRR + (IRR x MAP) + SOC + PCI + (GDDTb x EDD_{34})
Bean	10	1 + GDDTb + MAP + (GDDTb x MAP) + MAP^2 + $GDDTb^2$ + SOC + PCI + EDD_{30}
Cassava	13	1 + GDDTb + MAP + (GDDTb x MAP) + MAP^2 + $GDDTb^2$ + (IRR x MAP) + PCI + (GDDTb x EDD_{30}) + (IRR x EDD_{30})
Cotton	8	1 + GDDTb + MAP + (GDDTb x MAP) + MAP^2 + $GDDTb^2$ + (MAP x PCI) + SOC + PH + PCI + EDD_{26}
Grape	8	1 + GDDTb + MAP + MAP^2 + $GDDTb^2$ + SOC + PCI + EDD_{26}
Maize	8	1 + GDDTb + MAP + MAP^2 + $GDDTb^2$ + IRR + (IRR x MAP) + SOC + PCI + EDD_{26} + (GDDTb x EDD_{26}) + (IRR x EDD_{26})
Millet	8	1 + GDDTb + MAP + (GDDTb x MAP) + MAP^2 + $GDDTb^2$ + IRR + (IRR x MAP) + SOC
Oats	1	1 + GDDTb + MAP + (GDDTb x MAP) + MAP^2 + $GDDTb^2$ + PCI + (GDDTb x EDD_{34})
Oil palm	20	1 + GDDTb + MAP + MAP^2 + $GDDTb^2$ + IRR + (IRR x MAP) + (MAP x PCI) + (IRR x PCI) + PCI + EDD_{26}
Orange	0	1 + GDDTb + MAP + (GDDTb x MAP) + MAP^2 + $GDDTb^2$ + (MAP x PCI) + PH + PCI + EDD_{38} + (GDDTb x EDD_{38})
Potato	2	1 + GDDTb + MAP + (GDDTb x MAP) + MAP^2 + $GDDTb^2$ + IRR + (IRR x MAP + (MAP x PCI) + PCI + EDD_{34} + (GDDTb x EDD_{34})
Rapeseed	5	1 + GDDTb + MAP + MAP^2 + $GDDTb^2$ + (IRR x MAP) + SOC + PCI + EDD_{26} + (IRR x EDD_{26})
Rice	5	1 + GDDTb + MAP + (GDDTb x MAP) + MAP^2 + $GDDTb^2$ + IRR + (IRR x MAP) + PH + PCI + EDD_{30}
Rye	2	1 + GDDTb + MAP + (GDDTb x MAP) + MAP^2 + $GDDTb^2$ + (IRR x MAP) + SOC + EDD_{30} + (GDDTb x EDD_{30}) + (IRRxEDD_{30})
Sorghum	10	1 + GDDTb + MAP + (GDDTb x MAP) + MAP^2 + $GDDTb^2$ + IRR + (IRR x MAP) + (IRR x PCI) + SOC + EDD_{34} + (GDDTb x EDD_{34}) + (IRR x EDD_{34})
Soybean	8	1 + GDDTb + MAP + GDDTb x MAP + MAP^2 + $GDDTb^2$ + IRR + (IRR x MAP) + (IRR x PCI) + SOC + PH + (GDDTb x EDD_{34}) + (IRR x EDD_{34})

Crop	Base growing temp	Functional form of yield model
Sugar beets	2	1 + GDDTb + MAP + MAP^2 + $GDDTb^2$ + (IRR x MAP) + (IRR x PCI) + SOC + PH + PCI + EDD_{38} + (IRR x EDD_{38})
Sugarcane	12	1 + GDDTb + MAP + MAP^2 + $GDDTb^2$ + IRR + (IRR x MAP) + PCI
Sunflower	8	1 + GDDTb + MAP + (GDDTb x MAP) + MAP^2 + $GDDTb^2$ + IRR + (IRR x MAP) + PH + EDD_{26}
Sweet potato	8	1 + GDDTb + MAP + MAP^2 + $GDDTb^2$ + (MAP x PCI) + SOC + PCI + EDD_{38}
Tomato	5	1 + GDDTb + MAP + MAP^2 + $GDDTb^2$ + (MAP x PCI) + PH + PCI + EDD_{26} + (GDDTb x EDD_{26})
Wheat	0	1 + GDDTb + MAP + MAP^2 + $GDDTb^2$ + (IRR x MAP) + (MAP x PCI) + (IRR x PCI) + EDD_{26} + (GDDTb x EDD_{26})

c.4. Impact of climate change on yields

The impact of climate change on yields is estimated by using projections of future climate in the quantile regression model described above. More specifically, we use future predicted climate and temperature from the sixth Coupled Model Inter-Comparison Project (CMIP) https://pcmdi.llnl.gov/CMIP6/ as downscaled and bias-corrected by WorldClim (https://www.worldclim.org/data/cmip6/cmip6climate.html). We assume that the timeseries of variability around the daily mean as measured by the Berkeley Earth System Temperature dataset is identical in the future. Thus, the measure of EDD increases because the mean temperature increases; there is no simulated increase in variability. Soil variables are assumed to be static. Area equipped for irrigation is assumed to continue with current growth patterns following Gerber et al 2024 [main text ref. 93].

Future yields are calculated based on future climates predicted by each of 4 General Circulation Model (GCMs). These 4 GCMs were chosen for meeting two key criteria:

- Consistent with recommended solution for "hot model problem" (Transient Climate Response (TCR) between 1.4 and 2.2 °C).[112,113]

- Downscaled data exists for WorldClim2.1 future scenarios for 4 representative concentration pathways (RCPs)

These 4 sets of future yields are averaged to produce a mean predicted yield at 2030, 2050, 2070, and 2100. The 4 GCMs are shown in SM Table 10. We calculated yields under RCP 4.5.

Mechanics and/or key assumptions of calculating impacts of climate change on yield:

- Climate change had no impact on yields in 1985 (this allows us to use WorldClim historical data for a no-climate-change baseline).

- The impact of climate change on crop yields over the period from 1985-2018 is much smaller than the change in crop yields due to technical growth (improved agronomy and management) over this time. We are thus able to assume a negligible difference between the slope of the empirically observed yield time series from 1985-2018 (which includes impacts of climate change and technical growth) and the slope of the projected no-further-climate change yield time series (which includes technical growth).

- We calculate $\left.\frac{d(Yield)}{d(Climate)}\right|_{2030}$ for 2030 by comparing model yields using baseline weather data (1985) and projected data (2030). We use circa year 2000 model coefficients for this calculation because this is an era with relatively little climate change and relatively good crop data and environmental data.

- We assume $\left.\frac{d(Yield)}{d(Climate)}\right|_{2030}$ grows linearly from 1985 to 2030 thereby allowing us to estimate the impacts of climate change on 2018 yields $\left.\frac{d(Yield)}{d(Climate)}\right|_{2018}$ as $\left.\frac{33}{45}\frac{d(Yield)}{d(Climate)}\right|_{2030}$ and thus infer a change in yields relative to a no-climate-change counterfactual.

- The impact of climate change on yield growth subsequent to 2018 is then estimated to grow linearly to $\left.\frac{d(Yield)}{d(Climate)}\right|_{2030} - \left.\frac{d(Yield)}{d(Climate)}\right|_{2018}$ in 2030, with analogous growth to 2050 and beyond.

c.5 Benchmark test of model of climate change

We carried out two simple studies of the model of climate change impacts on yield to verify that the results are consistent with the literature. For the first, we compared impacts of a 1 degree increase in temperature on yields with those provided in the meta-analysis of Zhao et al (2017).[114] These results are shown graphically in SM Figure 7; results of the present model (after harvested-area weighted average aggregation across administrative units to the country level) are within the error bars for the majority of cases. The second test was to test the growth in yield impact with increasing temperature. We represent results of this test in SM Figure 8. Maize, wheat, and soybeans show increasing impact with temperature as expected. Rice does not – this is likely because this is an empirical model, and rice is an irrigated crop which can use evapotranspiration as a response mechanism to heat as historically observed. Figure 2A of Zhao et al. (2017) contains results for yield impact due to a 1-degree change in temperature from an ensemble of global statistical models for rice that is not significantly different from zero. Thus, this result for rice is not unexpected with this type of model.

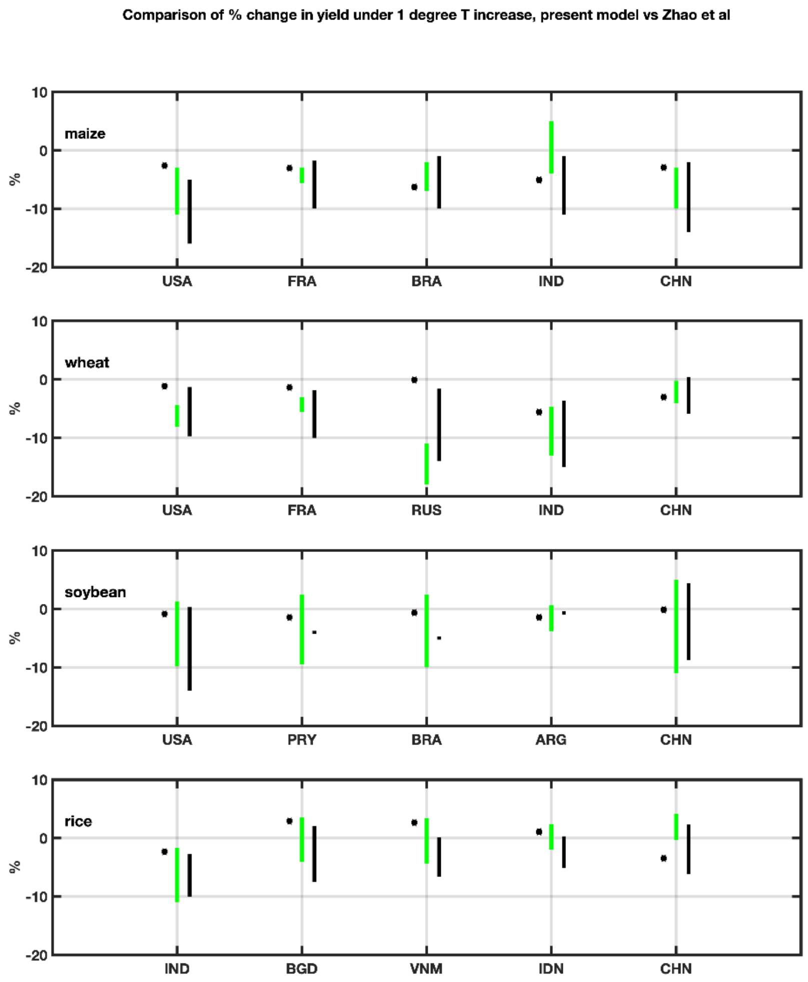

SM Figure 7. Comparison of change in yield under a 1 degree increase in temperature compared to meta-analysis results presented in Zhao et al. (2017). The * in the figures is the prediction of the current model, the green vertical line represents the confidence intervals around the statistical model result ensemble presented in Figure 3 of Zhao et al. (2017); the black vertical line represents the confidence interval around the ensemble of all model results presented in Figure 3 of Zhao et al. (2017). For Paraguay, Brazil, and Argentina in the soybean plot, no confidence interval was provided in Figure 3. Data was estimated from Figure 3 in Zhao et al. (2017).

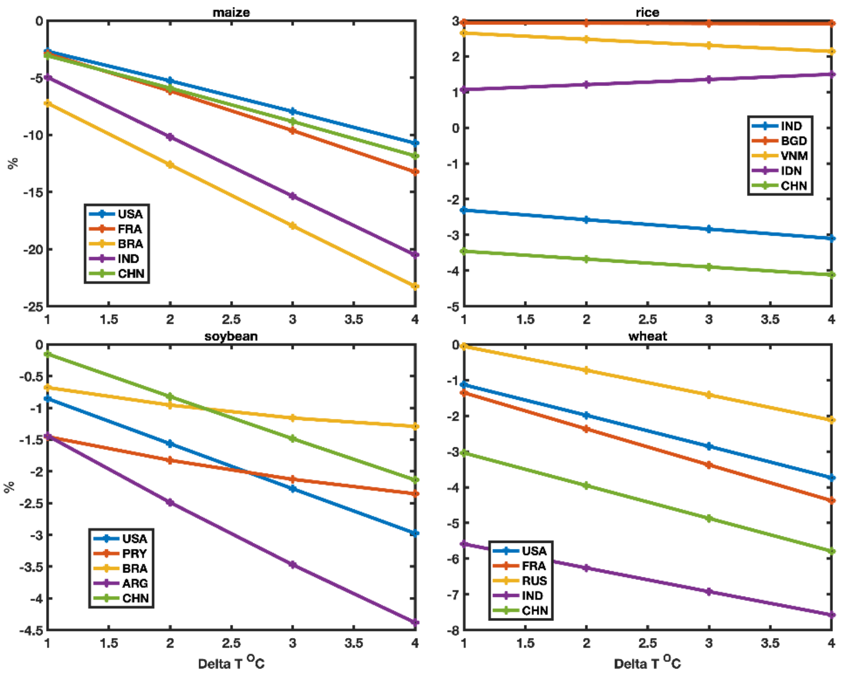

SM Figure 8. Behavior of impacts of temperature change on yield for the regions of Zhao et al. (2017). Generally linear increasing impact with temperature, as expected, is observed for maize, soybean, and wheat, but not for rice. These results are based on an empirical model, so non-linear impacts of future extreme temperatures are not expected to be present. The lack of increasing impact for rice may also be explained by the empirical nature of this model: as most rice is grown in flooded conditions, the mechanism of increased evapotranspiration may have averted decreases in yield due to increased temperature over the historical time span.

SM Table 10. General Circulation Models.

Model Name	Developing Country	Developing Institution	Link to Institution	TCR
ACCESS-ESM1-5	Australia	CSIRO	https://www.csiro.au/en/research/environmental-impacts/climate-change/ACCESS	1.91
BCC-CSM2-MR	China	Beijing Climate Center	http://bcc.ncc-cma.net	1.59
GISS-E2-1-G	USA	Goddard Inst for Space Studies	https://www.giss.nasa.gov/projects/gcm/	1.68
MIROC6	Japan	Model for Interdisciplinary Research on Climate 6	https://doi.org/10.5194/gmd-12-2727-2019	1.52

c.6 Converting projected yields of individual crops into a country-level projected yield

To project future cropland area, we converted individual crop, administrative unit-level yield projections at time t into one country-level yield projection at time t over several steps.

Step 1: We used data from FAO [main text ref. 5] to find a country's 2018 production (measured in tons) of each of the 23 crops with projected yields (SM Table 5). We then converted all

production measures from tons to kcals using conversion factors from Tilman et al. (2011) [main text ref. 13]. Finally, we summed across all 23 crops' 2018 production values measured in kcals to get a country's 2018 kcal production across the 23 focus crops. Let country i's 2018 kcal production across the 23 focus crops be given by $p_{i,23}$.

Step 2: We used data from FAO [main text ref. 5] to find a country's 2018 production (measured in tons) of all crops, not just the 23 focus crops. Then, we converted all production measures from tons to kcals as in Step 1 (if a crop does not have conversion factor then the crop was dropped) and summed across all crops' 2018 production values measured in kcals to get a country's total 2018 kcal production. Let country i's 2018 kcal production across all crops be given by p_i.

Step 3: We used data from FAO [main text ref. 5] to find a country's 2018 harvested hectare area across all crops included in Step 2. Let country i's 2018 harvested hectare area across all crops be given by h_i.

Step 4: Assuming harvested area of each crop in each administrative unit in country i remains fixed at 2018 levels, we estimated a country's 2050 and 2100 production (measured in tons) of each of the 23 focus crops. Then, we again converted all production measures from tons to kcals and summed across all 23 crops' 2050 and 2100 production values to get a country's 2050 and 2100 kcal production across the 23 focus crops. A country's future production across the 23 focus crops varied according to the set of future yields (tons per harvested hectare) we employed. Recall we have two sets of yields for 2050 and 2100: set 1) yield ceilings for each of the 23 focus crops assuming RCP 4.5 and set 2) linearly extrapolated yields for each of the 23 focus crops assuming RCP 4.5. Let country i's kcal production across the 23 focus crops in year t assuming future yield set w be given by $p_{i,23,t,w}$.

Step 5: We find country i's yield, measured in kcals per harvested hectare, at time t assuming future yield set w with the formula,

$$y_{i,t,w} = \frac{p_{i,23,t,w}}{p_{i,23}} \times \frac{p_i}{h_i} \qquad \text{(SM 8)}$$

Equation (SM 8) assumes that yields for all crops will increase at the same rate as the increase in yield among the 23 focus crops from 2018 to 2050 and 2100 and that the share (but not necessarily area) of harvested hectares across crops in each administrative unit is fixed at 2018 levels.

SM Table 11 presents the results for each country on: 1) observed 2018 yields (kcals harvested ha^{-1}); 2) projected 2050 and 2100 yields (kcals harvested ha^{-1}) with climate change (RCP 4.5) under each simulation scenario; and 3) estimated 2050 and 2100 yield ceilings (kcals harvested ha^{-1}) with climate change (RCP 4.5).

SM Table 11. Kcals per harvested hectare by country with climate change (RCP 4.5)

		(1)	(2)	(3)	(4)	(5)	(6)	(7)
**Country**	**Countries w/ acc. dev. in Acc. Dev and Eq. Dev. scenarios**	**Observed yield in 2018**	***BAU* and *Reduced crop demand in high-income countries* scenarios expected yields**		**Ceiling-level yields**		***Accelerated development in low-income countries* and *Equitable development* scenario expected yields**	
			**2050**	**2100**	**2050**	**2100**	**2050**	**2100**
Afghanistan	1	6,420,345	14,213,365	22,574,128	35,897,968	35,687,737	25,055,667	30,371,702
Angola	1	4,725,260	7,733,972	9,511,594	25,956,386	25,828,617	16,845,179	21,336,898
Albania	1	12,341,125	17,883,824	20,496,173	21,580,550	21,277,447	21,580,550	21,277,447
United Arab Emirates	0	16,514,545	1,609,574	1,894,308	1,956,011	1,937,079	1,609,574	1,894,308
Argentina	0	11,478,732	17,118,440	22,256,527	24,382,195	24,306,370	17,118,440	22,256,527
Armenia	1	7,886,745	17,093,109	23,523,782	30,673,296	30,332,391	23,883,202	27,107,797
Australia	0	6,932,878	8,391,404	9,899,895	24,913,014	24,697,277	8,391,404	9,899,895
Austria	0	19,110,691	23,284,655	26,962,779	30,708,144	30,490,230	23,284,655	26,962,779
Azerbaijan	1	8,893,719	12,889,600	18,662,232	22,395,871	22,198,328	17,787,497	19,992,912
Burundi	1	4,864,943	2,786,715	2,061,911	14,392,353	14,364,853	9,729,887	12,047,370
Belgium	0	26,302,999	29,874,956	27,348,278	34,077,082	33,992,704	29,874,956	27,348,278
Benin	1	6,129,693	7,226,232	9,477,666	19,094,872	19,115,287	13,160,552	16,137,920
Burkina Faso	1	3,432,849	3,440,261	4,256,370	9,661,023	9,588,169	6,865,700	8,226,935
Bangladesh	1	11,330,533	18,397,909	19,347,273	19,495,819	19,414,546	19,495,819	19,414,546
Bulgaria	0	14,417,951	16,565,158	21,211,859	22,035,954	21,895,256	16,565,158	21,211,859
Bosnia and Herzegovina	1	10,138,367	961,619	1,229,586	3,127,390	3,074,639	3,127,390	3,074,639
Belarus	1	9,665,247	17,048,084	23,454,367	25,741,148	25,564,776	21,394,616	23,479,696
Belize	1	9,548,728	9,598,182	11,257,483	21,918,726	21,869,200	19,097,452	20,483,326
Bolivia	1	7,398,871	7,213,389	9,187,147	18,660,763	18,567,699	14,797,724	16,682,712
Brazil	0	13,864,089	19,171,560	22,966,717	23,519,164	23,441,240	19,171,560	22,966,717
Brunei Darussalam	0	1,143,255	1,379,771	1,634,982	5,052,114	5,042,497	1,379,771	1,634,982
Bhutan	1	7,196,331	15,384,561	20,841,555	25,714,623	25,496,874	20,549,592	23,023,233
Botswana	1	2,156,021	893,528	996,318	23,068,513	22,575,607	11,981,020	17,278,314
Central African Republic	1	3,299,452	3,611,422	3,713,745	30,205,478	30,231,733	16,908,450	23,570,091
Canada	0	11,627,936	13,947,934	18,378,042	21,531,529	21,349,952	13,947,934	18,378,042
Switzerland	0	20,540,322	22,108,536	23,323,635	28,893,845	28,758,432	22,108,536	23,323,635
Chile	0	17,286,652	21,678,607	22,268,880	21,524,578	21,364,951	21,524,578	21,364,951
China	1	14,954,982	17,526,018	20,475,839	25,054,707	24,786,081	25,054,707	24,786,081
Cote d'Ivoire	1	3,209,776	2,048,175	2,696,540	8,553,309	8,934,233	6,419,545	7,676,889
Cameroon	1	6,802,053	6,046,079	4,561,497	17,378,826	17,494,376	13,604,098	15,549,237
Congo Dem. Rep.	1	5,650,455	5,632,228	6,517,063	22,450,875	22,636,443	14,041,551	18,338,997
Congo Rep.	1	8,587,769	11,893,380	16,191,435	18,514,290	18,760,977	17,175,518	17,968,247
Colombia	1	18,841,725	21,677,869	27,193,780	23,499,794	24,582,765	23,499,794	24,041,280
Costa Rica	0	21,922,713	23,389,410	20,250,791	37,395,777	37,616,589	23,389,410	20,250,791
Cuba	1	7,478,525	7,349,018	10,583,329	15,101,644	15,097,633	14,957,075	15,027,354
Czech Republic	0	17,062,279	24,356,774	26,385,079	27,298,198	27,132,553	24,356,774	26,385,079
Germany	0	20,243,605	28,791,834	29,569,826	29,769,462	29,642,443	28,791,834	29,569,826
Denmark	0	16,959,168	22,906,440	25,197,325	26,147,257	26,047,995	22,906,440	25,197,325
Dominican Republic	1	9,916,442	9,714,487	10,239,422	14,882,830	15,067,882	14,882,830	14,975,356
Algeria	1	6,994,384	8,419,650	12,076,657	21,816,613	21,589,118	15,118,132	18,353,625
Ecuador	1	14,526,812	22,402,360	28,218,472	33,561,360	34,096,267	29,053,577	31,574,922
Egypt Arab Rep.	1	19,022,527	28,532,588	29,484,065	31,034,549	30,954,681	31,034,549	30,954,681
Spain	0	10,035,764	11,066,374	14,278,463	17,276,268	17,048,463	11,066,374	14,278,463
Estonia	0	8,224,162	16,246,782	21,687,639	22,584,404	22,407,330	16,246,782	21,687,639

		(1)	(2)	(3)	(4)	(5)	(6)	(7)
**Country**	**Countries w/ acc. dev. in Acc. Dev and Eq. Dev. scenarios**	**Observed yield in 2018**	***BAU* and *Reduced crop demand in high-income countries* scenarios expected yields**		**Ceiling-level yields**		***Accelerated development in low-income countries* and *Equitable development* scenario expected yields**	
			**2050**	**2100**	**2050**	**2100**	**2050**	**2100**
Ethiopia	1	7,697,615	11,522,433	17,234,406	21,366,156	21,103,705	16,444,294	18,773,999
Finland	0	10,473,146	14,681,849	16,509,137	27,985,104	27,580,824	14,681,849	16,509,137
France	0	20,536,328	23,974,248	25,760,206	28,809,999	28,619,659	23,974,248	25,760,206
Gabon	0	5,283,874	4,668,441	5,202,880	26,694,731	26,883,330	4,668,441	5,202,880
United Kingdom	0	21,068,776	24,547,578	25,014,829	25,485,629	25,408,030	24,547,578	25,014,829
Georgia	1	5,329,030	4,620,421	4,312,150	29,623,491	29,250,450	17,121,956	23,186,203
Ghana	1	8,711,646	11,754,111	13,547,463	22,722,259	23,264,534	17,423,297	20,343,916
Guinea	1	4,265,956	3,678,322	3,536,151	24,001,567	24,578,059	13,839,944	19,209,002
Gambia The	1	2,701,025	2,585,638	1,898,094	16,826,526	16,978,135	9,706,082	13,342,109
Guinea-Bissau	1	3,742,464	3,741,108	3,846,383	12,521,297	12,733,676	8,131,203	10,432,439
Equatorial Guinea	0	5,079,243	6,429,772	8,100,621	18,436,867	18,555,179	6,429,772	8,100,621
Greece	0	8,500,596	9,827,287	11,351,608	17,326,737	17,139,976	9,827,287	11,351,608
Guatemala	1	13,908,636	16,225,698	17,529,709	29,014,436	28,978,463	27,817,293	28,397,878
Guyana	1	11,316,966	13,936,406	13,967,952	23,009,605	23,017,990	22,633,915	22,825,953
Honduras	1	14,207,653	15,212,135	15,570,207	23,938,293	24,486,168	23,938,293	24,212,230
Croatia	0	19,303,270	3,218,606	3,704,547	4,026,833	3,992,712	3,218,606	3,704,547
Haiti	1	2,626,890	2,985,144	3,205,541	20,531,135	20,411,644	11,758,139	16,084,891
Hungary	0	17,632,483	21,326,785	27,588,564	31,359,248	31,065,276	21,326,785	27,588,564
Indonesia	1	35,544,310	37,612,309	44,493,996	48,305,432	51,125,307	48,305,432	49,715,369
India	1	7,475,150	9,241,629	12,583,323	16,121,352	16,027,299	14,950,257	15,488,778
Ireland	0	22,346,413	32,608,406	32,353,490	26,133,427	26,034,600	26,133,427	26,034,600
Iran Islamic Rep.	1	7,157,738	8,830,063	10,907,615	23,405,599	23,223,429	16,117,831	19,670,630
Iraq	1	7,134,267	21,498,164	28,405,815	31,736,393	31,559,049	26,617,279	29,088,164
Israel	0	9,595,835	11,146,532	11,336,974	19,274,114	19,126,603	11,146,532	11,336,974
Italy	0	12,172,254	14,479,246	17,584,945	21,782,126	21,618,737	14,479,246	17,584,945
Jordan	1	7,929,146	9,258,684	8,934,520	17,957,468	17,686,104	15,858,292	16,772,198
Japan	0	15,284,060	16,949,851	18,396,502	21,181,472	21,119,950	16,949,851	18,396,502
Kazakhstan	0	4,554,910	5,618,549	8,015,866	25,401,618	25,289,313	5,618,549	8,015,866
Kenya	1	4,740,569	4,298,454	4,506,696	23,425,505	23,203,844	13,861,979	18,532,912
Kyrgyz Republic	1	9,544,294	11,374,046	14,677,698	28,496,460	28,280,650	19,935,253	24,107,952
Cambodia	1	10,474,236	17,305,502	20,078,132	20,515,238	20,453,081	20,515,238	20,453,081
Korea Rep.	0	13,994,691	15,775,053	16,223,020	18,039,609	18,004,080	15,775,053	16,223,020
Kuwait	0	32,510,963	67,331,700	65,649,103	37,993,725	37,549,243	37,993,725	37,549,243
Lao PDR	1	11,741,912	22,166,952	24,030,715	24,028,887	23,957,736	23,483,869	23,720,802
Lebanon	1	7,785,405	10,156,613	13,825,354	16,586,478	16,431,192	15,570,842	16,001,017
Liberia	1	4,375,256	5,646,183	6,441,568	19,247,568	19,709,004	12,446,875	16,077,940
Libya	1	2,722,752	2,413,187	2,018,462	21,597,013	21,410,263	12,005,100	16,707,682
Sri Lanka	1	7,138,486	9,380,371	12,395,267	15,472,709	15,464,418	14,276,947	14,870,683
Lithuania	0	10,255,775	17,799,663	21,025,155	21,684,237	21,566,969	17,799,663	21,025,155
Latvia	0	9,767,241	18,207,552	21,778,053	22,452,755	22,309,100	18,207,552	21,778,053
Morocco	1	6,659,762	7,965,439	11,782,195	22,083,156	21,854,142	15,024,298	18,439,220
Moldova	1	9,703,504	4,919,132	5,963,932	12,139,579	12,085,570	12,139,579	12,085,570
Madagascar	1	5,968,269	9,628,148	13,847,647	18,757,584	18,709,635	14,192,866	16,451,251
Mexico	0	11,695,419	14,438,992	19,311,863	29,791,993	29,464,363	14,438,992	19,311,863
Mali	1	5,236,099	5,980,469	7,862,763	9,700,179	9,551,965	9,700,179	9,551,965
Myanmar	1	7,139,645	11,697,587	15,846,658	16,432,038	16,413,314	14,279,307	15,346,310
Mongolia	1	4,385,875	3,862,628	5,930,919	19,650,482	19,563,953	11,756,555	15,660,254
Mozambique	1	3,075,019	4,030,887	5,396,138	21,907,988	21,711,567	12,969,438	17,340,502
Mauritania	1	3,737,070	2,910,911	2,561,716	5,499,932	4,566,429	5,499,932	4,566,429

		(1)	(2)	(3)	(4)	(5)	(6)	(7)
**Country**	**Countries w/ acc. dev. in Acc. Dev and Eq. Dev. scenarios**	**Observed yield in 2018**	***BAU* and *Reduced crop demand in high-income countries* scenarios expected yields**		**Ceiling-level yields**		***Accelerated development in low-income countries* and *Equitable development* scenario expected yields**	
			**2050**	**2100**	**2050**	**2100**	**2050**	**2100**
Malawi	1	6,691,239	10,431,552	13,424,980	26,837,537	26,542,217	18,634,544	22,588,381
Malaysia	0	72,990,543	116,609,502	131,972,228	107,730,616	114,280,241	107,730,616	114,280,241
Namibia	1	2,564,178	2,157,382	2,856,091	16,179,549	16,074,810	9,168,466	12,621,638
Niger	1	1,869,718	2,764,638	3,558,608	6,208,676	6,111,572	4,486,657	5,299,114
Nigeria	1	6,075,022	5,929,269	6,170,747	26,999,214	27,442,518	16,464,242	21,953,380
Nicaragua	1	8,856,156	9,972,758	9,952,049	26,984,401	26,964,887	18,478,579	22,721,733
Netherlands	0	29,676,438	35,635,216	35,549,512	35,434,284	35,384,933	35,434,284	35,384,933
Norway	0	9,335,879	13,512,008	14,189,384	24,609,358	24,415,065	13,512,008	14,189,384
Nepal	1	7,918,777	11,096,981	16,374,337	23,812,323	23,600,618	17,454,652	20,527,635
New Zealand	0	18,465,856	23,173,978	23,840,708	21,774,457	21,607,315	21,774,457	21,607,315
Oman	0	21,196,159	6,868,915	92,660,608	3,733,433	4,227,229	3,733,433	4,227,229
Pakistan	1	8,603,252	11,841,312	16,561,924	19,508,400	19,432,238	17,206,508	18,319,373
Panama	0	12,502,972	13,253,306	17,183,637	23,143,116	23,361,406	13,253,306	17,183,637
Peru	1	10,796,013	13,162,034	16,536,726	22,680,295	22,484,514	21,592,043	22,038,279
Philippines	1	8,743,587	12,150,870	17,576,818	22,106,945	22,101,686	17,487,180	19,794,433
Papua New Guinea	1	17,155,682	1,063,239	1,203,028	3,786,573	3,753,920	3,786,573	3,753,920
Poland	0	11,931,447	15,305,058	19,693,930	22,886,263	22,729,680	15,305,058	19,693,930
Korea Dem. People's Rep.	1	7,047,787	6,015,799	4,729,574	24,003,571	23,800,869	15,009,685	19,405,277
Portugal	0	6,348,099	7,093,475	7,456,847	9,646,492	9,567,876	7,093,475	7,456,847
Paraguay	1	12,206,825	14,416,624	18,841,704	24,790,367	24,762,665	24,413,657	24,588,161
Romania	0	17,093,216	16,776,705	23,879,882	30,294,094	30,055,662	16,776,705	23,879,882
Russian Federation	0	8,035,704	11,053,668	16,928,737	22,908,668	22,778,866	11,053,668	16,928,737
Rwanda	1	4,257,091	6,648,506	8,957,233	17,587,977	17,416,795	12,118,242	14,767,519
Saudi Arabia	0	16,931,447	7,205,757	7,170,288	7,072,476	6,974,881	7,072,476	6,974,881
Sudan	1	2,696,901	2,468,496	3,082,202	6,110,529	6,001,353	5,393,801	5,697,577
Senegal	1	5,252,925	5,161,857	6,532,052	9,522,912	9,536,477	9,522,912	9,529,694
Sierra Leone	1	3,972,046	7,447,686	10,860,796	18,637,002	18,835,182	13,042,344	15,938,763
El Salvador	1	8,137,706	11,011,594	14,228,630	22,260,978	22,161,488	16,636,286	19,398,887
Suriname	1	13,238,456	14,682,785	15,784,672	16,571,168	16,565,830	16,571,168	16,565,830
Slovak Republic	0	16,916,047	17,721,000	23,423,151	25,912,512	25,731,163	17,721,000	23,423,151
Slovenia	0	16,980,326	21,858,922	25,910,369	28,313,550	28,101,627	21,858,922	25,910,369
Sweden	0	12,408,033	19,479,102	22,053,265	26,573,553	26,375,739	19,479,102	22,053,265
Eswatini	1	11,247,152	9,826,541	8,816,074	27,087,091	26,841,860	22,494,292	24,668,076
Syrian Arab Republic	0	2,641,724	4,093,830	3,260,572	20,874,009	20,659,672	4,093,830	3,260,572
Chad	1	3,252,067	4,025,949	5,065,188	11,204,832	10,996,385	7,615,390	9,305,888
Togo	1	4,772,498	4,314,762	5,616,076	17,343,506	17,485,515	10,829,134	14,157,324
Thailand	1	12,807,923	18,183,411	22,736,032	22,347,893	22,688,423	22,347,893	22,518,158
Tajikistan	1	8,638,457	14,912,332	19,069,968	20,594,389	20,420,461	17,753,360	19,086,911
Turkmenistan	1	5,361,691	2,058,288	1,581,902	26,557,799	26,424,219	14,308,044	20,366,131
Tunisia	1	2,322,682	3,246,138	4,114,388	11,499,796	11,369,565	7,372,967	9,371,266
Turkey	0	9,959,360	12,810,984	16,086,095	22,970,599	22,757,090	12,810,984	16,086,095
Tanzania	1	4,829,920	4,554,102	5,329,682	23,403,585	23,254,323	13,978,843	18,616,583
Uganda	1	4,662,314	5,069,335	6,827,849	17,606,992	17,474,995	11,338,164	14,406,579
Ukraine	1	13,119,956	17,847,738	22,789,039	26,410,189	26,250,018	26,239,895	26,244,956
Uruguay	0	7,197,789	15,916,033	20,554,214	24,557,842	24,502,292	15,916,033	20,554,214
United States	0	21,122,724	27,327,371	29,301,925	31,940,369	31,734,709	27,327,371	29,301,925
Uzbekistan	1	11,142,036	16,407,487	16,596,757	17,139,878	17,042,670	17,139,878	17,042,670
Venezuela RB	0	9,363,173	19,039,281	24,113,564	37,423,332	37,642,466	19,039,281	24,113,564

		(1)	(2)	(3)	(4)	(5)	(6)	(7)
**Country**	**Countries w/ acc. dev. in Acc. Dev and Eq. Dev. scenarios**	**Observed yield in 2018**	***BAU* and *Reduced crop demand in high-income countries* scenarios expected yields**		**Ceiling-level yields**		***Accelerated development in low-income countries* and *Equitable development* scenario expected yields**	
			**2050**	**2100**	**2050**	**2100**	**2050**	**2100**
Vietnam	1	12,192,738	20,593,782	21,877,120	21,980,535	21,911,439	21,980,535	21,911,439
Yemen Rep.	1	2,583,789	3,355,260	2,952,079	16,831,501	16,647,692	10,093,381	13,370,536
South Africa	1	13,012,559	17,247,707	24,901,533	27,574,629	27,108,158	26,025,114	26,566,636
Zambia	1	7,530,850	10,625,583	14,897,250	26,544,514	26,247,183	18,585,048	22,416,116
Zimbabwe	1	4,131,540	1,067,572	358,347	33,137,746	32,458,028	17,102,659	24,780,343

c.7 Converting harvested hectares to cropland hectares

Harvested area and cropland area differ due to double-cropping, crop failure, etc. Using the yields from columns (2), (3), (6) and (7) of SM Table 11 in equation (SM 3) gives us harvested hectares in country *i* in year *t*. To convert harvested area to cropland area we use a fixed ratio of cropland area to harvested area. Region specific ratios are constructed using 2018 harvested area data and cropland area from FAO [main text ref. 5]. These regional ratios are reported in SM Table 12.

SM Table 12. Regional ratios of harvested area to cropland area.

**Low**	**Escaped low**	**Lower middle**	**Escaped lower middle**	**Upper middle and escaped upper middle**	**High**	**China**	**India**
0.89682	0.97242	0.85062	0.59113	0.920854	0.66564	1.22534	1.13122

d. Trade (Net exports)

We projected country-level net exports (exports – imports) in agricultural commodities in 2050 and 2100 using the Global Trade Analysis Project (GTAP) model version 7.0 and the GTAP Database (version 11) described in Aguilar et al (2022) [main text ref. 95] The GTAP model is one of the most frequently used models in the domain of applied trade analysis (Dellink et al. 2020).[115] The GTAP model is a computable general equilibrium model that follows standard microeconomic theory, namely that consumers maximize utility, producers maximize profits, and prices adjust so that supply equals demand in all markets. The GTAP model includes detailed international trade information, identifying bilateral trade relationships, data on tariffs, taxes and subsidies, and transport costs. We ran a disaggregated version of the model, including 33 sectors and 37 countries. We used 2017 as the base year for the GTAP analysis. To generate country-level net exports for 2050 and 2100 under the four scenarios (*BAU*, *Reduced per capita crop demand in the high-income countries*, *Accelerated development in the low-income countries*, and *Equitable development*) we used our country-level projections for 2050 and 2100 population, real annual per capita GDP, per capita daily kcal crop demand, and yield projections assuming RCP 4.5, for each scenario as input into the GTAP model. Given these inputs, the GTAP model predicted crop trade flows (in billions of real 2017 USD) between countries in 2050 and 2100 for each scenario.

Our model uses country-level net exports measured in calories (kcals) rather than dollars. We converted net trade in dollars by region in GTAP to net trade in kcals by country using the

following steps. First, we summed GTAP net exports in a region for a given year across all crops to get net exports for each GTAP region in each year (2017, 2050, 2100) under each scenario (for 2050 and 2100). GTAP crop categories are shown in SM Table 14.

SM Table 14. GTAP crop categories

Paddy rice
Wheat
Cereal grains nec
Vegetables, fruit, nuts
Oil seeds
Sugar cane, sugar beet
Plant-based fibers
Crops nec

Second, we estimated the following regression equation using data from 2018 to get an estimated relationship between country-level net exports measured in kcal and country-level net exports measured in dollars,

$$kcalNE_{i,2018} = \beta_0 + \beta_1 I[Low]_i + \beta_2 I[EscapeLow]_i + \beta_3 I[LowMid]_i$$

$$+\beta_4 I[EscLowMid]_i + \beta_5 I[UpMid]_i + \beta_6 I[High]_i + \beta_7 NE_{i,2017} + e_i \quad \text{(SM 10)}$$

where $kcalNE_{i,2018}$ is country i's net exports in kcals in 2018, $I[.]_i$ is an indicator variable that equals 1 if country i is in a particular income group and 0 otherwise, $NE_{i,2017}$ is country i's net exports in dollars in 2017 from GTAP, and e_i is an error term. In estimating equation (SM 8), we only included countries that are also single country GTAP regions. Some countries are part of a larger GTAP regions, and these countries were not included in the estimation. Countries that are not listed individually in GTAP but are part of a GTAP region are all relatively small net exporters without much influence on global trade patterns. SM Table 15 contains a crosswalk between GTAP regions and countries.

SM Table 15. Country – GTAP region crosswalk.

GTAP Region Number	GTAP Region Name	Country
1	AUS	Australia
2	NZL	New Zealand
3	XOC	Papua New Guinea
4	CHN	China
5	HKG	Hong Kong
6	JPN	Japan
7	KOR	Korea Rep.
8	MNG	Mongolia
9	TWN	Taiwan
10	XEA	Korea Dem. People's Rep.
11	BRN	Brunei Darussalam
12	KHM	Cambodia
13	IDN	Indonesia
14	LAO	Lao PDR

GTAP Region Number	GTAP Region Name	Country
15	MYS	Malaysia
16	PHL	Philippines
17	SGP	Singapore
18	THA	Thailand
19	VNM	Vietnam
20	XSE	Myanmar
21	AFG	Afghanistan
22	BGD	Bangladesh
23	IND	India
24	NPL	Nepal
25	PAK	Pakistan
26	LKA	Sri Lanka
27	XSA	Bhutan
28	CAN	Canada
29	USA	United States
30	MEX	Mexico
32	ARG	Argentina
33	BOL	Bolivia
34	BRA	Brazil
35	CHL	Chile
36	COL	Colombia
37	ECU	Ecuador
38	PRY	Paraguay
39	PER	Peru
40	URY	Uruguay
41	VEN	Venezuela RB
42	XSM	Guyana
42	XSM	Suriname
43	CRI	Costa Rica
44	GTM	Guatemala
45	HND	Honduras
46	NIC	Nicaragua
47	PAN	Panama
48	SLV	El Salvador
49	XCA	Belize
50	DOM	Dominican Republic
51	HTI	Haiti
52	JAM	Jamaica
53	PRI	Puerto Rico
54	TTO	Trinidad and Tobago
55	XCB	Cuba
56	AUT	Austria
57	BEL	Belgium
58	BGR	Bulgaria
59	HRV	Croatia
60	CYP	Cyprus
61	CZE	Czech Republic
62	DNK	Denmark
63	EST	Estonia
64	FIN	Finland
65	FRA	France
66	DEU	Germany

GTAP Region Number	GTAP Region Name	Country
67	GRC	Greece
68	HUN	Hungary
69	IRL	Ireland
70	ITA	Italy
71	LVA	Latvia
72	LTU	Lithuania
73	LUX	Luxembourg
74	MLT	Malta
75	NLD	Netherlands
76	POL	Poland
77	PRT	Portugal
78	ROU	Romania
79	SVK	Slovak Republic
80	SVN	Slovenia
81	ESP	Spain
82	SWE	Sweden
83	GBR	United Kingdom
84	CHE	Switzerland
85	NOR	Norway
86	XEF	Iceland
87	ALB	Albania
88	SRB	Serbia
89	BLR	Belarus
90	RUS	Russian Federation
91	UKR	Ukraine
92	XEE	Moldova
93	XER	Bosnia and Herzegovina
93	XER	Montenegro
94	KAZ	Kazakhstan
95	KGZ	Kyrgyz Republic
96	TJK	Tajikistan
97	UZB	Uzbekistan
98	XSU	Turkmenistan
99	ARM	Armenia
100	AZE	Azerbaijan
101	GEO	Georgia
102	BHR	Bahrain
103	IRN	Iran Islamic Rep.
104	IRQ	Iraq
105	ISR	Israel
106	JOR	Jordan
107	KWT	Kuwait
108	LBN	Lebanon
109	OMN	Oman
110	PSE	Palestinian Territory, Occupied
111	QAT	Qatar
112	SAU	Saudi Arabia
113	SYR	Syrian Arab Republic
114	TUR	Turkey
115	ARE	United Arab Emirates
116	XWS	Yemen Rep.
117	DZA	Algeria

GTAP Region Number	GTAP Region Name	Country
118	EGY	Egypt Arab Rep.
119	MAR	Morocco
120	TUN	Tunisia
121	XNF	Libya
122	BEN	Benin
123	BFA	Burkina Faso
124	CMR	Cameroon
125	CIV	Cote d'Ivoire
126	GHA	Ghana
127	GIN	Guinea
128	MLI	Mali
129	NER	Niger
130	NGA	Nigeria
131	SEN	Senegal
132	TGO	Togo
133	XWF	Gambia The
133	XWF	Guinea-Bissau
133	XWF	Liberia
133	XWF	Mauritania
133	XWF	Sierra Leone
134	CAF	Central African Republic
135	TCD	Chad
136	COG	Congo Rep.
137	COD	Congo Dem. Rep.
138	GNQ	Equatorial Guinea
139	GAB	Gabon
140	XAC	Angola
141	COM	Comoros
142	ETH	Ethiopia
143	KEN	Kenya
144	MDG	Madagascar
145	MWI	Malawi
146	MUS	Mauritius
147	MOZ	Mozambique
148	RWA	Rwanda
149	SDN	Sudan
150	TZA	Tanzania
151	UGA	Uganda
152	ZMB	Zambia
153	ZWE	Zimbabwe
154	XEC	Burundi
155	BWA	Botswana
156	SWZ	Eswatini
157	NAM	Namibia
158	ZAF	South Africa

In SM Table 16 we summarize estimated equation (SM 10).

SM Table 16. Estimated model (SM 8).

Variable	Estimated coefficient (Standard error)
Intercept	-1.6268 x 10^{14} *** (6.2075 x 10^{13})
Low	1.6162 x 10^{14} ** (6.4082 x 10^{13})
Escaped low	1.9692 x10^{14} *** (6.4012 x 10^{13})
Lower middle	1.6866 x 10^{14} ** (6.5275 x 10^{13})
Escaped lower middle	1.6465 x 10^{14} ** (6.4431 x 10^{13})
Upper middle and escaped upper middle	1.4962 x 10^{14} ** (6.4549 x 10^{13})
High	1.4385 x 10^{14} ** (6.3683 x 10^{13})
NE_{2017}	1.8706 x 10^{9} (1.1481 x 10^{9})
R^2	0.131

Notes. The other country category of India and China is the omitted country group. '***' indicates an estimated coefficient with a p-value <0.01, '**' 0.01 <p<0.05, and '*' 0.05<p <0.10.

Third, we used the estimated coefficients in equation (SM 8) to calculate each country's predicted net exports in kcals for 2018, $\widehat{kcalNE_{\iota,2018}}$, and the regression residual for each country,

$$\hat{e}_{i,2018} = kcalNE_{i,2018} - \widehat{kcalNE_{\iota}}_{,2018} \qquad \text{(SM 11)}$$

To calculate $\hat{e}_{i,2018}$ for country *i* that was part of a GTAP region we apportioned the region's net exports in 2017 to country *i* according to *i*'s share of the region's total 2017 GDP.

Finally, we predicted the net exports measured in kcals from country *i* in year *t* for a given economic scenario *s*, $\widehat{kcalNE_{\iota,t,s}}$, using the GTAP predicted net exports in dollars for that country in that year and scenario (given by $NE_{i,t,s}$), equation (SM 10)'s estimated regression coefficients, and the regression residual for country i (equation (SM 11)),

$$\widehat{kcalNE_{\iota,t,s}} = \hat{\beta}_0 + \hat{\beta}_1 I[Low]_i + \hat{\beta}_2 I[EscapeLow]_i + \hat{\beta}_3 I[LowMid]_i$$

$$+\hat{\beta}_4 I[EscLowMid]_i + \hat{\beta}_5 I[UpMid]_i + \hat{\beta}_6 I[High]_i + \hat{\beta}_7 NE_{i,t,s} + \hat{e}_{i,2018} \qquad \text{(SM 12)}$$

To calculate $NE_{i,t,s}$ for country *i* that was part of a GTAP region we apportioned the region's net exports in year *t* under scenario *s* according to *i*'s share of the region's total year *t* GDP.

SM Table 17 reports GTAP's sum of net exports in monetary terms across all countries for 2050 and 2100 under the different scenarios.

SM Table 17. Aggregate value of country-level exports (millions of real 2017 USD)

		2050		2100	
		**Aggregate exports**	**Relative change since base year**	**Aggregate exports**	**Relative change since base year**
Base year (2017)	190,074				
BAU / Reduced crop demand in high-income countries		381,057	2.005	1,503,069	7.908
Accelerated development in low-income countries and *Equitable development*		890,176	4.683	2,801,918	14.741

Notes: Assumes RCP 4.5. Relative change since base year: For example, 1.966 means a 96.6% gain.

d.1. Frictionless trade scenario

In our main results we solve equation (SM 3) for each country assuming trade between countries as estimated by GTAP (Figure 3 and 4.A). Alternatively, we calculated future cropland area across the globe under each scenario assuming an expanded trade scenario.

First, we found global crop demand of kcals in the years 2050 and 2100 under each scenario. Second, for each scenario, we found how many kcals the world would produce in 2050 and 2100 if harvested area in each country was fixed at 2018 levels but yields in each country reached their modeled 2050 and 2100 levels. Third, for each scenario and year, we subtracted global kcal crop demand from global kcal supply. If this generated number was greater than 0 then we found excess kcal supply in the modeled year and cropland around the world could be abounded relative to 2018 levels. Otherwise, if this balance was less than 0 then cropland around the world would have to be increased relative to 2018 levels to close the global kcal deficit.

To determine how much global cropland could be abandoned in the case of excess supply or how much global cropland had to be added in the case of excess demand under a scenario in year t we found the globe's kcal yield per harvested hectare in year t with,

$$y_t = \frac{\sum_{i=1}^{I} h_i y_{it}}{\sum_{i=1}^{I} h_i} \qquad \text{(SM 13)}$$

where h_i is country i's 2018 harvested hectare area across all crops and y_{it} is country i's projected yield in year t given the scenario (h_i was defined in section 3.c.6) We then divided the globe's harvested hectare excess or deficit by y^t to determine the harvested hectares around the world that could be abandoned or that had to be added in year t to balance global crop demand with global production. To convert harvested area change in year t to cropland area change in year t we used the globe's cropland area to harvested area ratio as observed in 2018 (SM Text 3.c.7, SM Table 12).

SM Text 4. FAOSTAT data

We use FAOSTAT products throughout this paper. Here we list the relevant FAOSTAT databases.

Cropland, 1961-2018. Production: Crops. Updated December 22, 2020. Accessed on December 24, 2020. http://fenixservices.fao.org/faostat/static/bulkdownloads/Population_E_All_Data_(Normalized).zip.

Harvested area, 1961-2018. Production: Crops. Updated December 22, 2020. Accessed on December 24, 2020. http://fenixservices.fao.org/faostat/static/bulkdownloads/Production_Crops_Livestock_E_All_Data_(Normalized).zip.

Harvested area, 2018. Production: Crops. Updated March 24, 2023 version. Accessed on June 1, 2024. http://fenixservices.fao.org/faostat/static/bulkdownloads/Production_Crops_Livestock_E_All_Data_(Normalized).zip. (This version of the dataset was used when projecting future yields.)

Cereal yield, 1961-2018. Production: Crops. Updated December 22, 2020. Accessed on December 24, 2020. http://fenixservices.fao.org/faostat/static/bulkdownloads/Production_Crops_Livestock_E_All_Data_(Normalized).zip.

Population, 1961-2018. Population: Annual population. Updated December 16, 2019. Accessed on July 1, 2020. http://fenixservices.fao.org/faostat/static/bulkdownloads/Population_E_All_Data_(Normalized).zip.

Agricultural import and export values, 1961-2018. Trade: Crops and livestock products. Updated February 9, 2021. Accessed on March 1, 2021. http://fenixservices.fao.org/faostat/static/bulkdownloads/Trade_Crops_Livestock_E_All_Data_(Normalized).zip.

Total nutrient Nitrogen, 1961-2018. Inputs: Fertilizer and Nutrients. Updated September 9, 2020. Accessed June 1, 2021. http://fenixservices.fao.org/faostat/static/bulkdownloads/Inputs_FertilizersNutrient_E_All_Data_(Normalized).zip.

Kcal crop production and demand, 1961-2018. Food and Agriculture Organization of the United Nations. 2020. Food Balance Sheets. Accessed on Dec 12, 2020. http://fenixservices.fao.org/faostat/static/bulkdownloads/Food_Balance_E_All_Data_(Normalized).zip.

SM Text 5. Country group rosters

SM Table 18. Country groups based on World Bank income group categories

Low-income countries	Afghanistan (AFG), Benin (BEN), Burkina Faso (BFA), Burundi (BDI), Central African Republic (CAF), Chad (TCD), Dem. Rep. Congo (COD), Eritrea (ERI), Ethiopia (ETH), Gambia (GMB), Guinea (GIN), Guinea-Bissau (GNB), Haiti (HTI), North Korea (PRK), Liberia (LBR), Madagascar (MDG), Malawi (MWI), Mali (MLI), Mozambique (MOZ), Nepal (NPL), Niger (NER), Rwanda (RWA), Sierra Leone (SLE), Somalia (SOM), Syria (SYR), Tajikistan (TJK), Tanzania (TZA), Togo (TGO), Uganda (UGA), Yemen (YEM)
Escaped low-income countries	Bangladesh (BGD), Bhutan (BTN), Cambodia (KHM), Equatorial Guinea (GNQ), Ghana (GHA), Guyana (GUY), Indonesia (IDN), Kenya (KEN), Laos (LAO), Lesotho (LSO), Mauritania (MRT), Myanmar (MMR), Nigeria (NGA), Pakistan (PAK), Solomon Islands (SLB), Sri Lanka (LKA), Sudan (SDN), Timor-Leste (TLS), Vietnam (VNM), Zambia (ZMB)
Lower-middle-income countries	Angola (AGO), Bolivia (BOL), Cameroon (CMR), Rep. of Congo (COG), Côte d'Ivoire (CIV), Djibouti (DJI), Egypt (EGY), El Salvador (SLV), Eswatini (SWZ), Honduras (HND), Kyrgyz Republic (KGZ), Moldova (MDA), Mongolia (MNG), Morocco (MAR), Nicaragua (NIC), Papua New Guinea (PNG), Philippines (PHL), Senegal (SEN), Tunisia (TUN), Ukraine (UKR), Uzbekistan (UZB), Vanuatu (VUT), Zimbabwe (ZWE)
Escaped lower-middle- income countries	Albania (ALB), Armenia (ARM), Azerbaijan (AZE), Belize (BLZ), Bosnia and Herzegovina (BIH), Botswana (BWA), Bulgaria (BGR), Chile (CHL), Colombia (COL), Costa Rica (CRI), Croatia (HRV), Cuba (CUB), Czech Republic (CZE), Dominican Republic (DOM), Dominica (DMA), Ecuador (ECU), Fiji (FJI), Georgia (GEO), Grenada (GRD), Guatemala (GTM), Jamaica (JAM), Jordan (JOR), Kazakhstan (KAZ), Lebanon (LBN), Malaysia (MYS), Mauritius (MUS), Mexico (MEX), Namibia (NAM), Paraguay (PRY), Peru (PER), Poland (POL), Slovakia (SVK), South Africa (ZAF), Thailand (THA), Turkey (TUR), Turkmenistan (TKM)
Upper-middle- and escaped upper-middle- income countries	Algeria (DZA), Antigua and Barbuda (ATG), Argentina (ARG), Barbados (BRB), Belarus (BLR), Brazil (BRA), Cyprus (CYP), Estonia (EST), Gabon (GAB), Greece (GRC), Hungary (HUN), Iran (IRN), Iraq (IRQ), South Korea (KOR), Latvia (LVA), Libya (LBY), Lithuania (LTU), Malta (MLT), Montenegro (MNE), New Caledonia (NCL), Oman (OMN), Panama (PAN), Portugal (PRT), Puerto Rico (PRI), Romania (ROU), Russian Federation (RUS), Serbia (SRB), Seychelles (SYC), Slovenia (SVN), Suriname (SUR), Trinidad and Tobago (TTO), Uruguay (URY), Venezuela (VEN)
High-income countries	Aruba (ABW), Australia (AUS), Austria (AUT), Bahamas (BHS), Bahrain (BHR), Belgium (BEL), Bermuda (BMU), Brunei Darussalam (BRN), Canada (CAN), Channel Islands (CHI), Denmark (DNK), Finland (FIN), France (FRA), Germany (DEU), Guam (GUM), Iceland (ISL), Ireland (IRL), Israel (ISR), Italy (ITA), Japan (JPN), Kuwait (KWT), Netherlands (NLD), New Zealand (NZL), Norway (NOR), Qatar (QAT), Saudi Arabia (SAU), Spain (ESP), Sweden (SWE), Switzerland (CHE), Taiwan (TWN), United Arab Emirates (ARE), United Kingdom (GBR), United States (USA)

The World Bank makes income-group classifications based on GNI per capita in US$ (Atlas methodology) thresholds that change year to year. See http://databank.worldbank.org/data/download/site-content/OGHIST.xls for the raw data.

Low-income countries were initially classified as low-income in 1987 and ended as low-income countries in 2018 or moved to low-income between 1987 and 2018 and were low-income in 2018.

Escaped low-income countries were classified as low-income initially and moved from low-income to lower-middle-income, upper-middle-income, or high-income between 1987 and 2018, and were lower-middle-income, upper-middle-income, or high-income in 2018.

Lower-middle-income countries were classified as lower-middle-income between 1987 and 2018 or classified as lower-middle-income initially and were lower-middle-income in 2018.

Escaped lower-middle-income countries were classified as lower-middle-income initially and moved from lower-middle-income to upper-middle-income or high-income between 1987 and 2018 and were upper-middle-income or high-income in 2018.

Upper-middle-income and escaped upper-middle-income countries were classified as upper-middle-income between 1987 and 2018 or classified as upper-middle-income initially and were upper-middle-income in 2018 or were classified as upper-middle-income initially and moved to high-income between 1987 and 2018.

High-income countries: were classified as high-income between 1987 and 2018.

China and India not included in these categories as they were analyzed separately.

We include all countries listed in SM Table 18 along with India and China in the analyses for Figures 1 – 4 and Table 1 in the main text with the following exceptions.

Figure 1: includes countries that had an uninterrupted series of data between 1961 and 2018 for the variable in question. Countries that had border changes between 1961 and 2018 are not included. Countries with border changes include those in the former Soviet Union (Armenia, Azerbaijan, Belarus, Estonia, Georgia, Kazakhstan, Kyrgyz Republic, Latvia, Lithuania, Moldova, Russian Federation, Tajikistan, Turkmenistan, Ukraine, Uzbekistan), several countries in Eastern Europe (Bosnia and Herzegovina, Croatia, Czech Republic, Montenegro, Serbia, Slovakia, Slovenia), several countries in Africa (Eritrea, Ethiopia, Sudan), and several other countries (Belgium, Qatar, United Arab Emirates). Several countries were missing data on cereal yields (generally countries with very little agriculture): Aruba, Antigua and Barbuda, Bahrain, Bermuda, Channel Islands, Djibouti, Equatorial Guinea, Iceland, Kuwait, Seychelles. Aruba and Channel Islands were also missing crop demand data.

Table 1: The regressions presented in Table 1 are estimated with data from countries that have uninterrupted series of dependent and independent variable data between 1961 and 2016 and have population exceeding one million people. If a country that otherwise has an uninterrupted series of data only has a population exceeding one million people for some years, then they were only included in those years when population exceeding one million (for example, if population exceeded one million between 2010 to 2016, then only its 2010 to 2016 data is included in the regression equation). Countries not included in Table 1 are Armenia, Aruba, Antigua and

Barbuda, Azerbaijan, Barbados, Belarus, Belgium, Bermuda, Bhutan, Bosnia and Herzegovina, Brunei, Channel Islands, Croatia, Czech Republic, Djibouti, Dominica, Equatorial Guinea, Eritrea, Estonia, Ethiopia, Fiji, Georgia, Grenada, Guam, Guyana, Iceland, Kazakhstan, Kyrgyz Republic, Kuwait, Laos, Latvia, Lithuania, Malta, Mauritius, Moldova, Montenegro, New Caledonia, North Korea, Puerto Rico, Qatar, Russian Federation, Serbia, Seychelles, Slovakia, Slovenia, Solomon Islands, Sudan, Surinam, Taiwan, Tajikistan, Turkmenistan, Ukraine, United Arab Emirates, Uzbekistan, Vanuatu.

In Figure 2, trendlines only include data from the countries in that income group that had an uninterrupted series of data between 1961 and 2018 for both kcals produced and kcals consumed. Countries not included in Figure 2 are Armenia, Aruba, Azerbaijan, Bahamas, Bahrain, Belarus, Belgium, Belize, Bermuda, Bosnia and Herzegovina, Channel Islands, Croatia, Czech Republic, Eritrea, Estonia, Ethiopia, Georgia, Guam, Kazakhstan, Kyrgyz Republic, Kuwait, Laos, Latvia, Lithuania, Moldova, Montenegro, Qatar, Russian Federation, Serbia, Seychelles, Slovakia, Slovenia, Sudan, Taiwan, Tajikistan, Turkmenistan, Ukraine, United Arab Emirates, Uzbekistan.

In Figures 3 and 4, we included countries with available data for 2017/2018 kcal crop demand levels; 2018 cropland area; 2018 harvested hectares; 2018, 2050 and 2100 GDP per capita values; 2050 and 2100 kcal crop demand levels; 2018, 2050, and 2100 population estimates; and 2050 and 2100 crop yield estimates. Countries not included in Figures 3 and 4 are: Aruba, Antigua and Barbuda, Bahamas, Bahrain, Bermuda, Barbados, Channel Islands, Cyprus, Djibouti, Dominica, Eritrea, Fiji, Grenada, Guam, Iceland, Jamaica, Lesotho, Malta, Mauritius, Montenegro, New Caledonia, Puerto Rico, Qatar, Solomon Islands, Seychelles, Somalia, Serbia, Taiwan, Timor-Leste, Trinidad and Tobago, Vanuatu.

SM Text 6. Factors driving demand and supply for crops in the US 1866-2020.

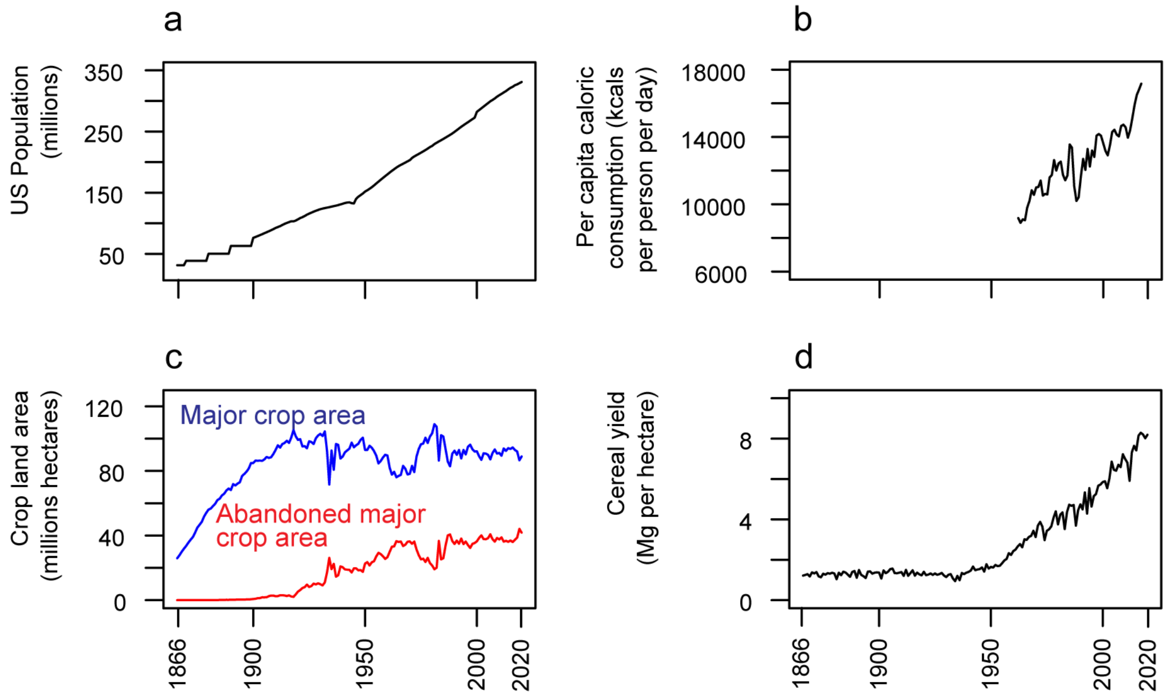

SM Figure 9. Factors driving demand and supply for crops in the US 1866-2020. (a) Population (millions), (b) Per capita crop demand (all uses; kcal cap^{-1} day^{-1}), (c) Harvested cropland area for major crops (million ha), (d) Cereal yield (tons per ha). Crop demand includes direct human consumption of crops, crop waste, crops used in production of livestock, aquaculture, biofuels, sweeteners, and other plant-based products. Cropland for major crops is the sum of harvested hectares of maize, wheat, barley, oats, rye, sorghum, soybeans, potatoes, and cotton. For SM Figure 9c, we calculate abandoned cropland for each state in each year by taking the difference between the maximum annual cropland ever observed in that state and the cropland observed in that year. We then sum abandoned cropland across all states in each year. Cereals in the cereal yield trend include barley, maize, oats, sorghum, rice, rye, and wheat; cereal yield each year is the sum of cereal production in tons divided by the total harvested area. Data sources: (a) Texas State Library (2020)[116], (b) FAO [main text ref. 5], (c, d) USDA NASS (2021)[117], also see Fig. 4 in Yu and Lu (2018)[118].

USDA data on harvested hectares extend back to 1866 but only include harvested but not planted hectares. Thus, the definition of harvested cropland area used in SM Figure 9c is not the same as used elsewhere in the paper. Cropland area in the US expanded rapidly between 1866 and 1920 (SM Figure 9c). Between 1920 and World War II falling exports and the Great Depression reduced per capita demand and lowered agricultural prices, leading to a slight decline in cropland despite continued population growth (Sumner et al. 2010, Goldstein 1989)[119,120]. After World War II, greater consumption of grain-fed livestock and other animal products and food waste caused the daily demand of US consumers to double from 1950 levels to ~16,000 kcal of crop production per capita per day by 2020 (SM Figure 9.b). This amount vastly exceeds the per capita daily consumption of food needed for a healthy diet (2,200 kcal/person/day). Since 1950, the large increase in total demand for crop kcal (greater per capita demand + increased

population + increased exports) was met by higher crop yields rather than cropland expansion (SM Figure 9c, d). Government programs expanded markets for crop-based animal feed, biofuels and sweeteners, and used payments to pull land from production (e.g., Soil Bank Program, Conservation Reserve Program). These policies helped to maintain agricultural crop prices and farmer incomes but greatly limited the reduction in cropland area that otherwise would have occurred as US yields increased 4-fold from 1950 to 2020 (SM Figure 9d).

Note that focusing on net cropland area masks considerable land clearing and abandonment in the US from 1866 to 2018 (red line in SM Figure 9c): US cropland has been ~90 million ha for the past century, while ~40 million ha were abandoned from agriculture, and ~40 million ha were cleared as production shifted to the Midwest from other parts of the country. Although abandoned croplands eventually return to a more natural state, this process takes decades to centuries. Therefore, even relatively constant total acreage may mask continued destruction and fragmentation of natural habitats.

SM Text 7. Gross and net transition to and from cropland between 1992 and 2018 in each country group

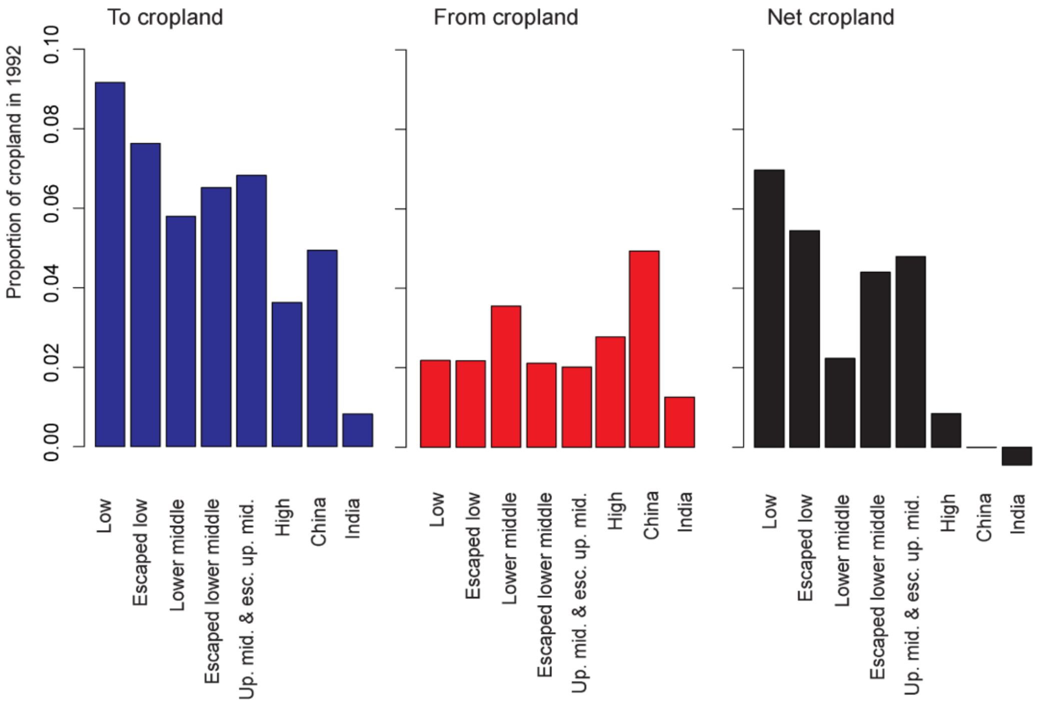

SM Figure 10. Gross and net transition to and from cropland between 1992 and 2018 in each country group. a. Transition to cropland. b. Abandonment of cropland. c. Net change in aggregate cropland. Data are based on satellite coverage of land use over time [main text ref. 79]. Land in the former USSR is included in this analysis. Satellite cropland is the aggregate of the land cover categories 'Cropland, rainfed', 'Cropland, rainfed, herbaceous cover', 'Cropland, rainfed, tree or shrub cover', and 'Cropland, irrigated or post-flooding.'

SM Text 8. Percentage change in country-level (A) population, (B) daily kcal crop demand per capita, (C) cropland area, and (D) cereal yield by country group

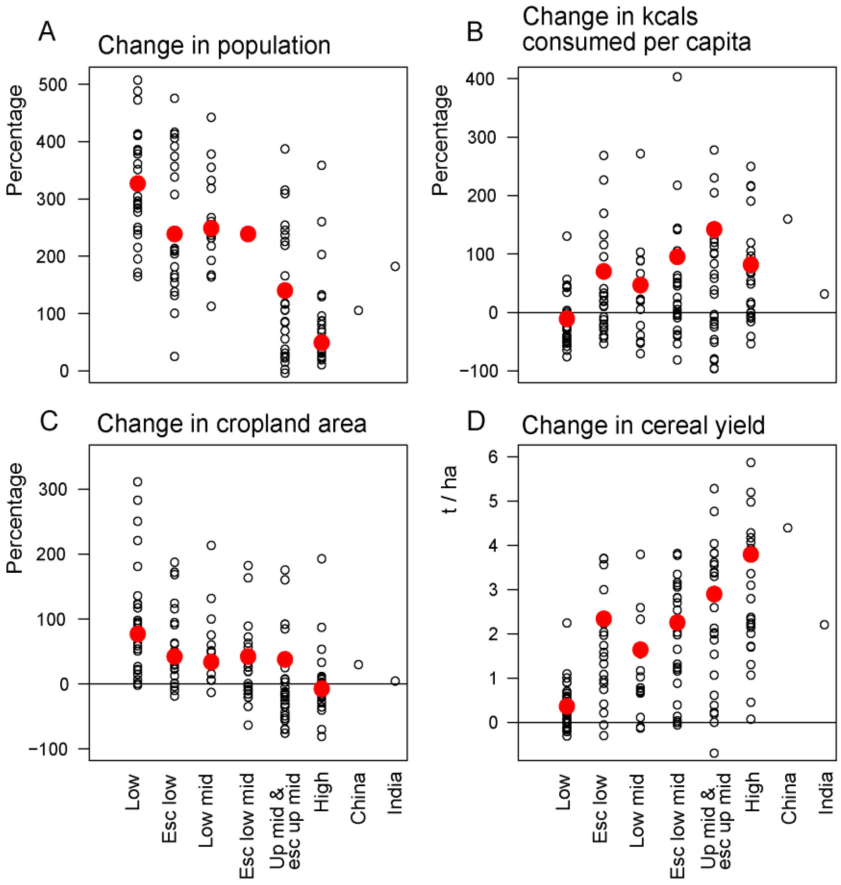

SM Figure 11. 1963-1965 to 2016-2018 percentage change in country-level (A) population, (B) daily kcal crop demand per capita, (C) cropland area, and (D) cereal yield organized by country group. We used a three-year average for each time series endpoint to reduce the impact of outliers on percentage calculations. Each unfilled plot point represents results for a single country. The red plot points give the income group's overall percentage change. The countries that are excluded from Figure 1 are excluded from this figure. Data sources: FAO [main text ref. 5]

Supplementary Materials and Methods References

96. Fontagné, L.,*et al.*, MaGE 3.1: Long-term macroeconomic projections of the world economy. *Internat. Econ.* **172**, 168-189 (2022).
97. Rennert, K., *et al.*, Comprehensive evidence implies a higher social cost of CO_2. *Nature* **610**, 687–692 (2022).
98. Lobell, D.B., *et al.*, The critical role of extreme heat for maize production in the United States. *Nature Climate Change* **3**, 497-501 (2013).
99. Meinshausen, N., Ridgeway, G. Quantile regression forests. *J. Mach. Learn. Res.* **7** (2006).
100. Guilpart, N., *et al.*, Data-driven projections suggest large opportunities to improve Europe's soybean self-sufficiency under climate change. *Nat. Food* **3**, 255–265 (2022).
101. World Bank. 2021. *The Changing Wealth of Nations 2021: Managing Assets for the Future*. (World Bank, Washington, DC, 2019).
102. Monfreda, C., *et al.*, Farming the planet: 2. Geographic distribution of crop areas, yields, physiological types, and net primary production in the year 2000. *Global Biogeochemical Cycles*. **22**(1) (2008).
103. Fick, S.E., Hijmans, R.J., WorldClim 2: New 1-km spatial resolution climate surfaces for global land areas. *Int. J. Climatology* **37(12)**, 4302–4315 (2017).
104. Oliver, J.E., Monthly precipitation distribution: A comparative index. *The Professional Geographer* **32(3)**, 300–309 (2010).
105. Hengl, T., *et al.*, SoilGrids250m: Global gridded soil information based on machine learning. *PLoS One* **12(2)**, e0169748 (2017).
106. FAO/IIASA/ISRIC/ISS-CAS/JRC, 2009. Harmonized World Soil Database (version 1.1). FAO, Rome, Italy and IIASA, Laxenburg, Austria.
107. Batjes, N., ISRIC-WISE derived soil properties on a 5 by 5 arc-minutes global grid, version 1.0 (ISRIC—World Soil Information, Wageningen, 2006).
108. Mueller, N.D., *et al.*, Closing yield gaps through nutrient and water management. *Nature* **490**, 254–257 (2012).
109. Portmann, F.T. *et al.*, MIRCA2000—Global monthly irrigated and rainfed crop areas around the year 2000: A new high-resolution data set for agricultural and hydrological modeling. *Glob. Biogeochemical Cycles* **24(1)**, GB101 (2010).
110. Siebert, S., *et al.*, A global data set of the extent of irrigated land from 1900 to 2005. *Hydrol. Earth Syst. Sci.* **19**, 1521–1545 (2015).
111. Evenson, R.E., Fuglie. K.O., Technology capital: the price of admission to the growth club. *J. Productivity Anal.* **33,** 173-190 (2010).
112. Hausfather, Z., *et al.*, Climate simulations: recognize the 'hot model' problem. *Nature* **605**, 26–29 (2022).
113. Nijsse, F.J.M.M., Cox, P.M., Williamson M.S., Emergent constraints on transient climate response (TCR) and equilibrium climate sensitivity (ECS) from historical warming in CMIP5 and CMIP6 models. *Earth Syst. Dyn.* **11**, 737–750 (2020).
114. C. Zhao, C., *et al.*, Temperature increase reduces global yields of major crops in four independent estimates. *Proc. Natl. Acad. Sci. U. S. A.* **114**, 9326–9331 (2017).
115. Dellink, R., van der Mensbrugghe, D., Saveyn, B., Shaping baseline scenarios of economic activity with CGE models: Introduction to the special issue. *J. Global Econ. Anal.* **5**(1), 1 (2020).

116. Texas State Library. *United States and Texas Populations 1850-2017*. Updated January 8, 2020. Accessed on Feb.1, 2021. https://www.tsl.texas.gov/ref/abouttx/census.html.
117. U.S. Department of Agriculture, National Agricultural Statistics Service. 2021. *Quick Stats. Agriculture Survey Data, 1866 – 2020*. Accessed on Feb.1, 2021. https://quickstats.nass.usda.gov/
118. Yu, Z., Lu, C., Historical cropland expansion and abandonment in the continental U.S. during 1850 to 2016. *Glob. Ecol. Biogeogr.* **27**, 322–333 (2018).
119. Sumner, D.A., Alston, J.M., Glauber, J.W., Evolution of the economics of agricultural policy. *Am. J. Agric. Econ.* **92**, 403–423 (2010).
120. Goldstein, J., The impact of ideas on trade policy: The origins of US agricultural and manufacturing policies. *Int. Org.* **43**, 31-71 (1989).
